# Directed Evolution of RecA Variants with Enhanced Capacity for Conjugational Recombination

**DOI:** 10.1371/journal.pgen.1005278

**Published:** 2015-06-05

**Authors:** Taejin Kim, Sindhu Chitteni-Pattu, Benjamin L. Cox, Elizabeth A. Wood, Steven J. Sandler, Michael M. Cox

**Affiliations:** 1 Department of Biochemistry, University of Wisconsin-Madison, Madison, Wisconsin, United States of America; 2 Department of Medical Physics, University of Wisconsin-Madison, Madison, Wisconsin, United States of America; 3 Department of Microbiology, University of Massachusetts-Amherst, Amherst, Massachusetts, United States of America; National Cancer Institute, UNITED STATES

## Abstract

The recombination activity of *Escherichia coli* (*E*. *coli*) RecA protein reflects an evolutionary balance between the positive and potentially deleterious effects of recombination. We have perturbed that balance, generating RecA variants exhibiting improved recombination functionality via random mutagenesis followed by directed evolution for enhanced function in conjugation. A *recA* gene segment encoding a 59 residue segment of the protein (Val79-Ala137), encompassing an extensive subunit-subunit interface region, was subjected to degenerate oligonucleotide-mediated mutagenesis. An iterative selection process generated at least 18 *recA* gene variants capable of producing a higher yield of transconjugants. Three of the variant proteins, RecA I102L, RecA V79L and RecA E86G/C90G were characterized based on their prominence. Relative to wild type RecA, the selected RecA variants exhibited faster rates of ATP hydrolysis, more rapid displacement of SSB, decreased inhibition by the RecX regulator protein, and in general displayed a greater persistence on DNA. The enhancement in conjugational function comes at the price of a measurable RecA-mediated cellular growth deficiency. Persistent DNA binding represents a barrier to other processes of DNA metabolism *in vivo*. The growth deficiency is alleviated by expression of the functionally robust RecX protein from *Neisseria gonorrhoeae*. RecA filaments can be a barrier to processes like replication and transcription. RecA regulation by RecX protein is important in maintaining an optimal balance between recombination and other aspects of DNA metabolism.

## Author Summary

The genetic recombination systems of bacteria have not evolved for optimal enzymatic function. As recombination and recombination systems can have deleterious effects, these systems have evolved sufficient function to repair a level of DNA double strand breaks typically encountered during replication and cell division. However, maintenance of genome stability requires a proper balance between all aspects of DNA metabolism. A substantial increase in recombinase function is possible, but it comes with a cellular cost. Here, we use a kind of directed evolution to generate variants of the *Escherichia coli* RecA protein with an enhanced capacity to promote conjugational recombination. The mutations all occur within a targeted 59 amino acid segment of the protein, encompassing a significant part of the subunit-subunit interface. The RecA variants exhibit a range of altered activities. In general, the mutations appear to increase RecA protein persistence as filaments formed on DNA creating barriers to DNA replication and/or transcription. The barriers can be eliminated via expression of more robust forms of a RecA regulator, the RecX protein. The results elucidate an evolutionary compromise between the beneficial and deleterious effects of recombination.

## Introduction

A given segment of chromosomal DNA may be subjected to repair, transcription, replication, and recombination, some or all of these processes occurring within a single cell cycle. Each of these processes poses real or potential molecular problems for the others, and many sources of genome instability lie at the interfaces [1–4]. The interface between replication and transcription has been the subject of numerous studies [5–7]. The role of collisions between replication forks and transient template discontinuities created by DNA repair events in the creation of double strand breaks is now well appreciated, as is the importance of recombinational DNA repair of those breaks [8–16]. In contrast, the potentially negative effects of recombinational DNA repair on other aspects of DNA metabolism have not been systematically investigated. The study described here is based on the following premise: (a) recombination systems can have negative impacts on DNA metabolism; (b) for that reason, recombinases such as RecA have not evolved to promote their characteristic DNA pairing and strand exchange activities optimally, but instead reflect an evolutionary compromise between the positive and negative effects of recombination; (c) substantial increases in recombinase functionality should be possible; and (d) since they were not selected during evolution, increases in recombinase functionality may have deleterious effects on cellular DNA metabolism.

The bacterial RecA recombinase plays a key role in recombinational DNA repair in *E*. *coli* [11, 17–23]. Inactivation of recombination functions results not only in DNA repair defects, but also in more general genomic instability such as stalled or collapsed replication forks [12, 14, 16, 24–29]. The major activity of RecA protein in homologous genetic recombination reaction is the promotion of DNA strand invasion and strand exchange [30–36]. RecA functions as a helical nucleoprotein filament, which assembles on and dissociates from DNA in several steps [37–41], and displays a diversity of conformations and dynamics [42, 43]. RecA has several additional cellular functions. Its filaments, when formed on DNA, act as a coprotease to promote the autocatalytic cleavage of the LexA repressor leading to induction of the bacterial SOS response [44–54]. RecA also activates the mutagenic DNA polymerase V, a function induced late in the SOS response [45, 55–59]. In this final role, RecA again acts as a coprotease to promote the autocatalytic cleavage of the UmuD subunit [60–62], and acts itself as an essential subunit of the final activated enzyme [63–67].

*In vitro*, RecA protein is a DNA-dependent ATPase and promotes DNA strand exchange reactions that mimic its presumed roles in vivo [18–20, 36, 68–70]. RecA filament formation on single-stranded DNA (ssDNA) begins with a slow nucleation step, followed by rapid 5′ to 3′ extension. RecA filament extension occurs predominantly at the 3′-proximal end. When ATP is hydrolyzed, RecA monomers disassemble primarily at the 5′-proximal end [71–75]. For the strand exchange reaction, RecA filaments form on ssDNA first, followed by pairing with a homologous double-stranded DNA (dsDNA) [30, 35, 76]. Exchange of strands ensues and can encompass thousands of DNA base pairs as ATP is hydrolyzed [34, 36, 70, 77–79].

Whereas recombination is necessary for double strand break repair and can produce genetic advantages via conjugation, recombination can also lead to genomic damage; e.g., by aberrant elimination of genomic segments due to recombination between repeated sequences. In principle, the RecA protein can also harm cells and contribute to genome instability in at least three other ways. First, RecA could inappropriately induce the SOS response, with its accompanying cell division regulation and mutagenesis, when it is not needed [80, 81]. Second, if RecA filaments were not efficiently removed from the DNA when no longer needed, replication and/or transcription could be inhibited. Third, a replication fork or transcription bubble collision with a branched DNA segment undergoing recombinational DNA repair could have a myriad of deleterious consequences. A multi-level regulatory system thus constrains and directs productive RecA-mediated recombination processes in the cell [25]. Several regulatory proteins, RecFOR [72, 82–87], RecX [88–92], DinI [91, 93, 94], RdgC [95], PsiB [96], DinD [97], RadA [80], and UvrD [98–100] are known to be involved in the regulation of RecA activity through interactions with the RecA nucleoprotein filament. An understanding of this regulation network is one prerequisite to an optimal *in vivo* harnessing of the recombination capacity of RecA.

A particular focus of the current study is the *E*. *coli* RecX protein, a conserved and well-characterized RecA regulator expressed from a gene located immediately downstream of the *recA* gene in *E*. *coli* and many other bacteria. The RecX protein is a negative regulator of RecA, required to overcome deleterious effects of overexpression of RecA protein [101–104]. Deletion of the *recX* gene does not cause a clear phenotype in *E*. *coli* [105], but overexpression reduces induction of the SOS response [106]. *In vitro*, the *E*. *coli* RecX (EcRecX) protein inhibits RecA-mediated ATPase and strand exchange activities [106]. The EcRecX protein binds deep within the major helical groove [107] and blocks the extension of a RecA filament by capping its 3′-proximal end while allowing filament disassembly to proceed at the 5′-proximal end [88]. The RecX protein from the bacterium *Neisseria gonorrhoeae* (NgRecX) exhibits a substantially more robust inhibition of RecA protein [90]. Instead of simply capping the growing filament end, the NgRecX appears to create breaks in the filament and increase the number of disassembling ends [90]. In spite of the often modest phenotypes seen in *E*. *coli* strains lacking *recX* function, interaction with RecX protein may be one of the key mechanisms that regulate the stability and recombination function of RecA nucleoprotein filaments in most bacteria.

The bacterial RecA protein was first identified from the analysis of mutagenized colonies of an F^-^ culture that were unable to form recombinants after conjugation with an Hfr strain [21]. Conjugational recombination is thus a classic function of RecA that helps define its recombination potential. During bacterial conjugation, once the mating pairs are established, rolling circle replication initiates at the F^-^ plasmid *oriT* site. Then a nascent single stranded Hfr DNA with a 5′ end enters the F^-^ recipient where it provides a template for lagging strand synthesis [108]. Transfer of DNA ceases at random points and leaves a linear double-stranded Hfr DNA fragment with a leading end and a single stranded overhang of variable length at the distal 3′ end because of the failure to complete synthesis of the complementary strand [109]. In the recipient, genetic crossovers promoted by RecA protein and auxiliary proteins integrate the Hfr fragment into the host genome. Two or more recombination events may occur concurrently or divergently, and the size of the integrated Hfr DNA varies.

The *recA* gene has the capacity to evolve to meet extraordinary cellular challenges such as radiation damage [110–117]. Specific amino acid changes at the subunit-subunit interface produce RecA variants that promote higher levels of conjugational recombination [118]. These results are the basis for the hypothesis that RecA has not evolved for optimal recombination function but instead for an optimal balance between the necessary and potentially damaging consequences of recombination within a particular environmental context. We therefore set out to explore the limits of RecA recombination function. Conjugational recombination has been employed as a selection for RecA variants with the potential to generate higher numbers of crossovers between unlinked genetic markers. Based on the demonstrated functional enhancement observed in some RecA variants with alterations at the subunit-subunit interface [118], our first effort has focused on this region. In this study, we demonstrate a facile generation of RecA variants that enhance recombination function. The results begin to define some of the resulting biochemical changes that potentially contribute to the enhancement and highlight some of the constraints placed on RecA function *in vivo*. We also explore the sometimes deleterious cellular consequences of these functional enhancements.

## Results

### Overview

Three questions are addressed below in three successive sections. (1) Can increases in RecA functionality be obtained? This involves a directed evolution experiment focused on increasing conjugational recombination function. (2) What changes in RecA activity give rise to the functional enhancements? A thorough *in vitro* characterization of several RecA variants is carried out to address this question. (3) What are the cellular consequences of a functionally enhanced RecA recombinase? Cell growth deficiencies associated with RecA functional enhancements are documented and explained.

### Directed evolution of RecA proteins with enhanced function in conjugation

#### Rationale

**Fig 1:**
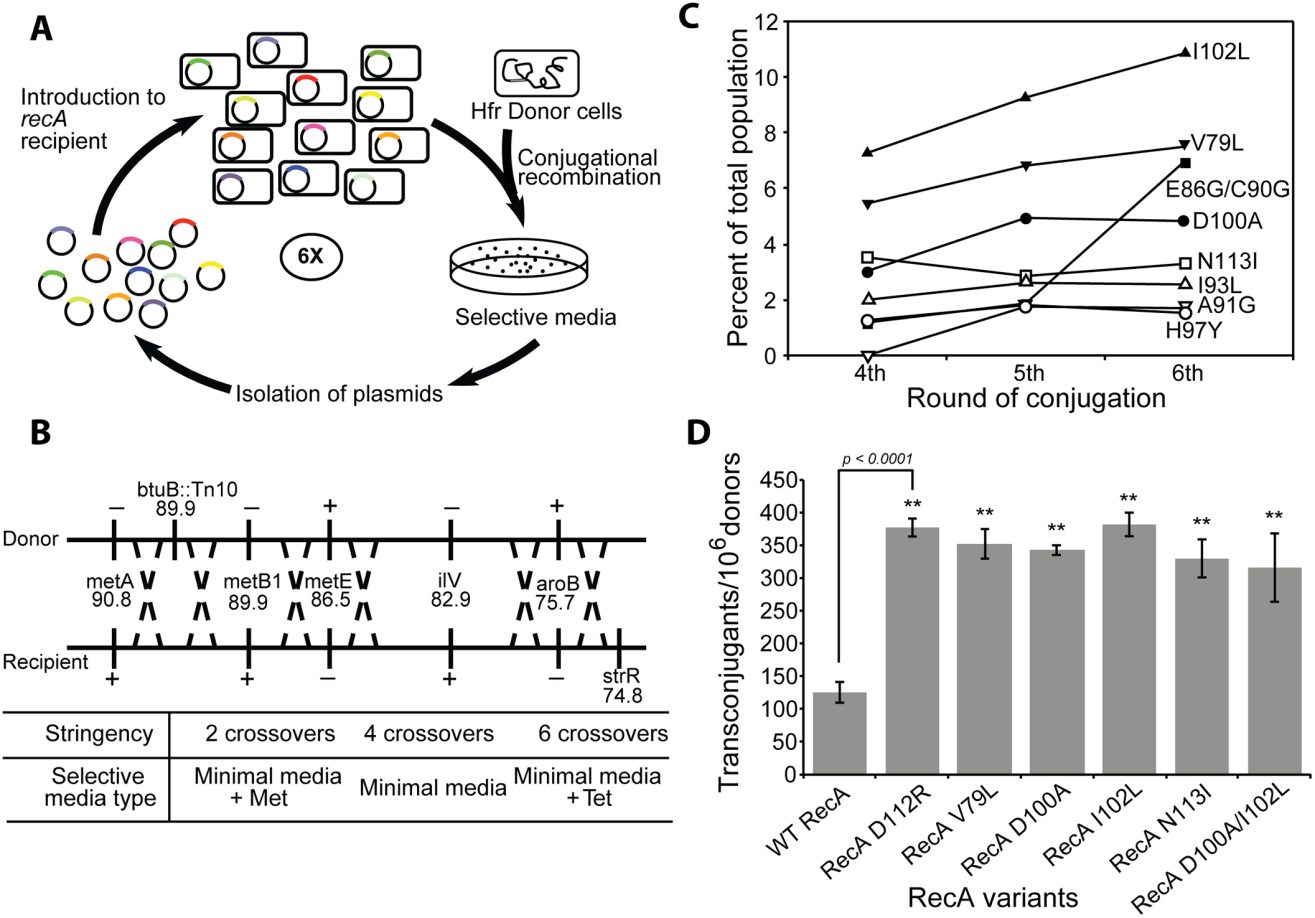
The directed evolution trials. (A) Scheme for directed evolution of RecA variants with improved functionality in conjugational recombination. (B) Genetic markers used in the conjugation trials. A series of genetic markers were added or deleted from Hfr donor and recipient strains in order to establish scalable stringency of the conjugation trials. Depending on the types of growing media, the number of crossovers required to produce transconjugant can be varied from 2 to 6. (C) The appearance of prominent RecA protein variants in the selected libraries after the 4th, 5th and 6th cycle of selection in the first directed evolution trial. Results are based on deep sequencing of each plasmid pool purified after the respective cycles. The 8 most prominent RecA variants are summarized. (D) Improved yield of transconjugants utilizing several prominent RecA variants from the directed evolution trials. The individual plasmid bearing a particular mutated *recA* gene expressing one of four RecA variants (RecA V79L, RecA D100A, RecA I102L and RecA N113I) was introduced into new *recA* ^-^ recipient cells. Conjugational recombination efficiency was measured for each RecA variant. The previously reported RecA D112R [118] and wild type RecA protein were tested as well, In one case, two mutations were introduced in combination (RecA D100A/I102L) to test for synergistic effects.

The first goal of this study was to systematically generate RecA protein variants with an enhanced capacity to promote conjugational recombination and to explore the limits of RecA function in bacteria. We used degenerate oligonucleotide synthesis to mutagenize a 59 codon stretch of the *recA* gene, with the segment size constrained by commercial limitations on oligonucleotide length. The region from codon 79 to 137 was chosen as a target in this initial study. This region of the gene encodes an expansive part of the subunit-subunit interface (residues 111–140) [119], and includes codons whose alteration demonstrably improves the recombination potential of the RecA protein [118]. To screen this region comprehensively, a plasmid library was constructed containing expressed *recA* genes with every possible single nucleotide change within that 59 codon region. Note that due to the structure of the genetic code [120, 121], single nucleotide changes do not provide access to every possible amino acid substitution, but our library should include over a third of them. The plasmid library is hosted by the F^–^ recipient for a conjugational cross designed to be restrictive. That F^–^ recipient includes an inactivated chromosomal *recA* gene, so that the only RecA protein expressed in a given cell is contributed by a library plasmid. The selection is scalable: using different media, we can require two, four, or six conjugational crossovers to produce a viable recombinant. Recipients hosting plasmids expressing nonfunctional RecA proteins simply die because they do not recombine in the selectable traits. Every recombinant colony that appears has a plasmid expressing an active RecA protein, either wild type or variant. The plasmids present in recombinants from the first cross are collected, isolated, and introduced into a fresh culture of *recA* ^–^ recipient cells. By carrying out the selection iteratively, RecA variants with an increased capacity for conjugational recombination should increase as a percentage of the overall population with successive cycles. Since the successful RecA-expressing plasmids are isolated after each cycle and installed in new recipient hosts, background genomic mutations that might contribute to recombination proficiency are eliminated. The overall scheme is similar in rationale to the SELEX method for generating RNAs that bind to a particular ligand [122–125], and is illustrated in Fig 1A.

#### *E*. *coli* RecA library construction

The synthesis of degenerate DNA molecules using different molar ratios of four nucleotides in mixtures was technically limited to 100 nucleotides. To extend the range of *recA* gene mutagenesis, two degenerate oligonucleotides were annealed in tandem to a 200 nucleotide complementary oligonucleotide with wild-type sequence. To synthesize the mutagenized oligos, each nucleotide addition included either 98.5% or 99% of the correct nucleotide, plus 0.5% or 0.33% of each of the incorrect nucleotides. This permitted a random mutagenesis of the codons encoding residues 79 to 137 calculated to maximize the presence of single nucleotide substitutions and minimize multiple base substitutions within the resulting library. The library was characterized in several ways. More than 70% of the plasmids expressed wild type RecA protein. Single mutants comprised 16.6%, with the remainder double, triple, and quadruple mutants (S1A Fig). The library included a total of 27,500 colonies with plasmids of all types, and approximately 4565 with single *recA* mutations. A Monte Carlo-based analysis indicated that there was a greater than 90% confidence that the library included all possible single mutations in the targeted region (S1B Fig)

#### Selection of active RecA variants with enhanced recombination activity

The conjugational cross used for selection featured a range of markers (*metA*, *metB1*, *metE*, *TetR*, *ilv*, *aroB*, and *StrR*) spread over a 16 min chromosomal region. Multiple genetic exchanges are required to generate a recombinant bacterium that can grow on a particular selective medium (Fig 1B). An Hfr-recipient pair should undergo 6 recombinational crossovers during conjugation to generate the cell able to grow on minimal media supplemented with tetracycline and streptomycin. The number of required crossovers can be reduced to 4 or 2 by selecting transconjugants on minimal media or methionine supplemented minimal media, respectively. In this system, recombination stringency is thus scalable. In the experiments requiring 4 crossovers, and recipients with plasmids expressing the wild type RecA protein, about 100–150 recombinant colonies per 1,000,000 donors were generated. When higher recombination stringency was applied (requiring 6 crossovers), the number of transconjugant colonies was reduced to 10–50.

The first conjugation carried out with recipient cells hosting library plasmids utilized the 4 crossover requirement. The recombination frequency was 100 transconjugants per 1,000,000 donors, similar to the experiment with recipients expressing the wild type RecA protein. More than 2,000 recombinant colonies were generated and combined in a pool. The population of *recA* gene-bearing plasmids was isolated and introduced into a new batch of *recA* ^–^ recipient cells for the next conjugation. At a second round of conjugation, recombination frequency increased nearly five folds, resulting in approximately 500 recombinants per 1,000,000 donors. A total 10,000 colonies was generated, combined and the plasmids isolated as before. For subsequent cycles, the recombination stringency was increased to 6 crossovers and this continued for an additional 4 consecutive cycles of selective conjugation. The frequency of successful recombination substantially declined to 40 transconjugants per 1,000,000 donors after the third cycle due to the increased stringency. More than 800 colonies were harvested. Successful recombination increased to 75 transconjugants after the fourth and fifth cycles, declining again to 40 per 1,000,000 donors after the sixth cycle. An archival sample of the *recA* plasmid population was taken and stored after each cycle. From each of 4th, 5th and 6th round of conjugation, 20–30 recombinant colonies were selected at random for sequencing to get an approximate mutation pattern. Based on this profile, *recA* genes on the archived plasmid population pools from 4th, 5th and 6th round of conjugation were subjected to deep Illumina sequencing that allowed for detection of any mutation present as more than 0.5% of the total population.

The ratio of sequences expressing wild type RecA protein versus sequences expressing mutant RecA protein from 4th, 5th and 6th round of conjugation are presented in S2 Fig. In the initial cell library, the wild type *recA* gene represented more than 70% of the population. Wild type genes represented only 25.7% after the 6th round of conjugation. The portion of *recA* sequences with mutations increased from 60.5% (4th conjugation) to 69.2% (5th conjugation) and reached 74.3% after the 6th round of conjugation. These results infer the presence of RecA variants that are more proficient at promoting the recombination events required to generate transconjugants in this cross. Sequences from 4th, 5th and 6th round of conjugation were translated to determine specific amino acid changes arising with prominence in the population. All mutations arising above the 0.5% threshold in the selection are shown in S3 Fig. The populations for the eight most prominent variants after the 4th, 5th, and 6th cycles of conjugation are summarized in Fig 1C. All amino acid changes which emerged from the 4th round of conjugation (V79L, E86G, C90G, I93L, H97Y, D100A, I102L and N113I) were consistently found through the 6th round of conjugation. The portion of the population with each of these mutations continued to increase with successive cycles except H97Y and N113I. The V79L and I102L single changes were the most prominent after every conjugation cycle, representing 7.5% and 10.8% of the population, respectively, after the 6th cycle of conjugation. The E86G/C90G double mutant was less than 2% of the population until the 5th conjugation cycle, but remarkably increased to 7.0% after the 6th round conjugation (S3 Fig).

Beginning with the original library, the entire selection procedure was repeated to determine reproducibility. A total of 7 cycles of selective conjugation were carried out in this 2nd selection experiment. Amino acid changes found after 5th, 6th and 7th round of conjugation in this second selection experiment are shown in S4 Fig. The I93L variant was most prominent after the seventh cycle (13.0% of the population), and the A131G variant was the second most prominent at 8.9%. The V79L and I102L changes that dominated the first experiment were 3.7% and 0.5% of the population, respectively. Importantly, most of the RecA variants selected for in the second experiment were also found in the first experiment (S5 Fig). The exceptions were limited to two variants (D100A and G136R) found only in the first experiment, and three others (A81V, A104V and D110A) found only in the second. The results suggest that the selection protocol is near saturation with respect to identifying RecA variants with improved recombination capacity in this region of the *recA* gene.

To confirm that the increased prominence of certain mutations after selection can be considered as a gain of recombination function in these mutant proteins, conjugational recombination frequencies were directly tested for the most prominent RecA variants and compared to the wild type protein. Each plasmid harboring prominent mutations after the 6th round of conjugation was separately introduced to new recipient cells and the conjugation recombination assay requiring 4 crossovers was carried out. In this test, the recipient expressing wild type RecA protein was able to generate 125.3 ± 15.4 recombinant colonies per 10^6^ donors (Fig 1D). The RecA D112R variant, a previously reported hyper RecA mutant protein [118], was also tested as reference and produced 377.3 ± 14.5 recombinants in this conjugational test. RecA variants selected from our conjugation screening exhibited similar recombination activities. The recipient with RecA V79L, D100A, I102L and N113I variants made 352.8 ± 23.3, 342.8 ± 7.3, 382.3 ± 18.0 and 330.2 ± 28.8 transconjugants, respectively, per 10^6^ donors. The mutations are not necessarily additive in their effects. We constructed a double mutant protein that combined mutations D100A and I102L. The double mutant protein generated 316.0 ± 53.0 recombinant colonies (Fig 1D), somewhat less than each of the single mutants alone.

### *In vitro* characterization of RecA protein mutants with enhanced functionality

#### Enhanced capacities of RecA protein variants to hydrolyze ATP and displace SSB on circular ssDNA

From the first directed evolution trial, the most prominent three mutants from the 6th cycle of conjugation, I102L, V79L and the E86G/C90G double mutant, were selected for *in vitro* characterization. Each of these RecA variant proteins was expressed and purified. The characterization reflects standard RecA activities. The focus here is on activities that helped to define the deleterious effects of the RecA variants in section 3 below.

**Fig 2:**
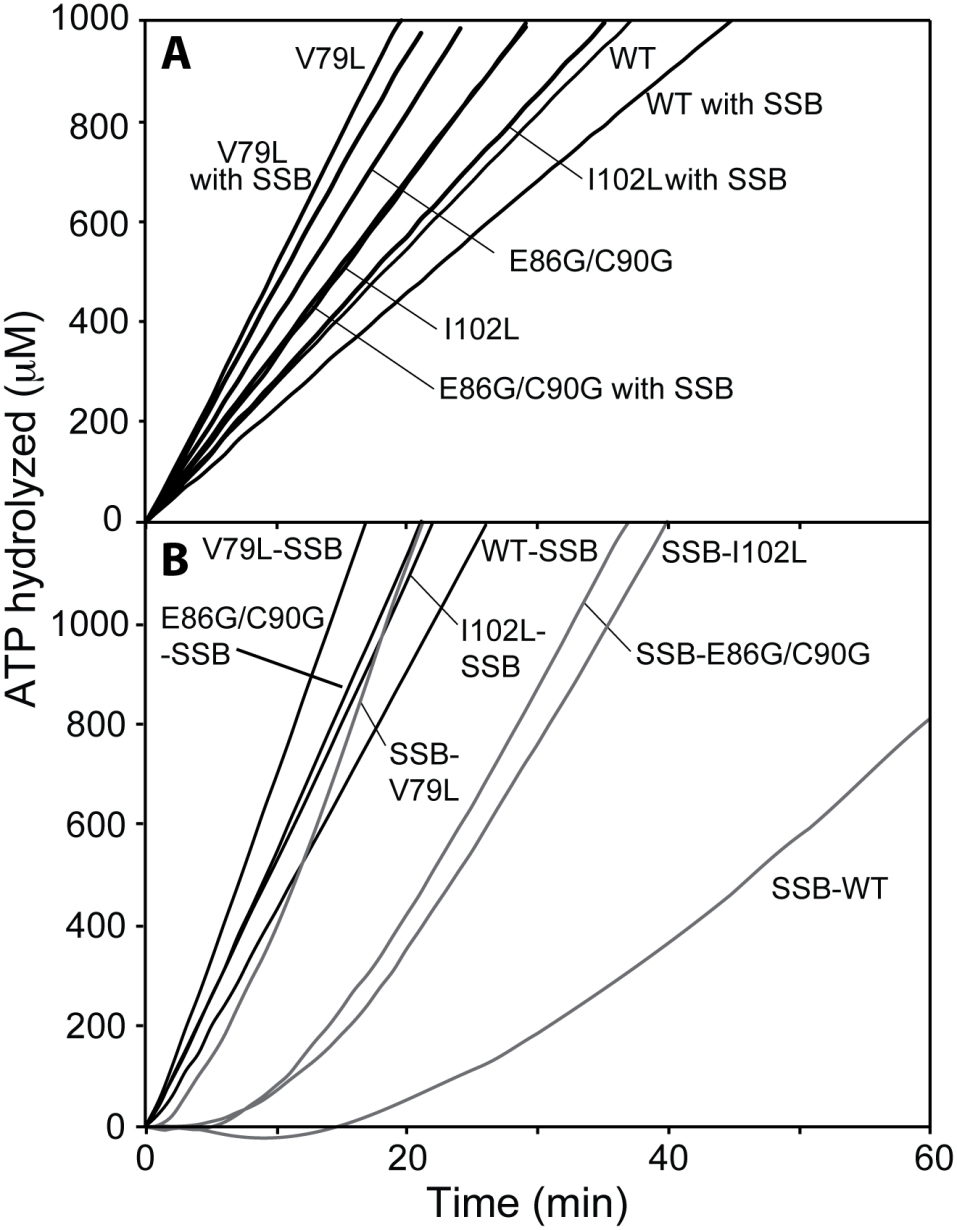
DNA-dependent ATPase activity of selected RecA variant proteins. (A) ATPase activity of RecA variant proteins on (dT)_60_ oligomeric DNA and inhibition effects of SSB protein on re-nucleation of RecA variant. Reactions contained 1.7 μM RecA variant, 5.1 μM (dT)_60_ oligomeric DNA and 3 mM ATP. The sub-saturating concentration of each RecA variant was incubated with DNA for 10 min and ATP was added to initiate the reaction. In a separate set of experiment, SSB protein was added (0.1 μM) at 10 min after the reaction was initiated to inhibit the re-assembly of free RecA variant in the solution (denoted by “with SSB”), and ATP hydrolysis was again monitored. (B) ATPase activity of RecA variant proteins on M13mp18 cssDNA and effects of SSB protein. Reactions contained 3 μM RecA variant, 5 μM M13mp18 ssDNA, 0.5 μM SSB, and 3 mM ATP. The *E*. *coli* SSB protein was added with ATP at 10 min after RecA variants filaments assembled (SSB listed second), or incubated with DNA for 10 min prior to the addition of RecA variant (SSB listed first).

To provide some baselines and an initial comparison, DNA binding and its associated ATP hydrolytic activity was examined with a short oligonucleotide cofactor, (dT)_60_ (Fig 2A). When bound to short linear single strands, RecA filaments are in a dynamic equilibrium in which new RecA filaments are constantly being formed, and existing ones are disassembling [18, 126]. In this reaction, a stoichiometric amount (1.7 μM) of each RecA protein was used with 5.1 μM of the (dT)_60_ oligomer. The apparent *k*_cat_ for the wild type, RecA I102L, V79L and E86G/C90G proteins was 15.7 ± 0.1, 19.5 ± 0.7, 30.1 ± 0.6 and 24.0 ± 0.3 min^–1^, respectively. In all cases, the measured *k*_*cat*_ values were lower than those measured on circular single stranded DNA (cssDNA) with single stranded DNA binding protein (SSB) added last, a number we generally associate with the state where all or nearly all of the DNA is bound. With the short oligos, the dynamics of filament assembly and disassembly prevent full binding of the DNA under these conditions. However, rates of ATP hydrolysis associated with (dT)_60_, as a fraction of the apparent maximum rate, were greater for the RecA variants than for the wild type. The wild type, RecA I102L, V79L and E86G/C90G proteins promoted ATP hydrolysis at 54.7, 57.0, 68.3, and 68.4%, respectively, of the rates seen on cssDNA. The results suggest that more of the DNA is bound by the RecA variants than by the wild type RecA protein under these conditions.

On short single-stranded DNA, SSB protein can bind to (dT)_60_ oligomer as an existing filament disassembles and suppresses re-nucleation of new RecA filaments. When 0.1 μM of SSB protein was added after RecA filaments had been formed, the apparent *k*_cat_ of wild type RecA protein declined by 27.6% to 11.4 ± 0.8 min^−1^. The reductions in ATP hydrolysis (and by inference of overall DNA binding) were less for the RecA variants: 16.2% and 15.6% for the RecA I102L and E86G/C90G mutant proteins and only 10.9% for RecA V79L. The apparent attenuation in SSB inhibition for the RecA variants could be explained either by an improvement in re-nucleation on SSB-coated ssDNA or a reduction in filament disassembly, or both.

We then examined the filament formation and ATP hydrolytic activities of the selected RecA protein variants on cssDNA (Fig 2B). M13mp18 cssDNA was first incubated with the different RecA proteins to allow them to nucleate and form filaments on the DNA, followed by addition of ATP and SSB. When added after RecA, SSB protein promotes RecA protein filament extension by melting secondary structure in the DNA. Full filaments are formed (reflecting complete or nearly complete binding of the available DNA), and the resulting rates thus better represent the intrinsic ATPase activity of a given variant. RecA I102L, V79L and E86G/C90G exhibited apparent *k*_cat_ values of 34.2 ± 0.6, 44.1 ± 0.8, and 35.1 ± 1.3 min^−1^, respectively, again displaying the higher ATPase levels associated with the RecA variants and providing a baseline for additional studies. These rates were consistently higher than the rates of wild type RecA protein, at 28.7 ± 0.2 min^−1^, a value consistent with previous findings [89, 127–133]. The increases in the intrinsic capacity of the RecA variants to hydrolyze ATP are unusual for this protein. In these studies, we measure apparent *k*_cat_ which reflects the observed rate of ATP hydrolysis divided by the concentration of available RecA protein binding sites (assuming a RecA subunit binds to 3 nucleotides). When the apparent *k*_cat_ values decline in other situations, as in the continuing studies below, this generally reflects a decrease in the fraction of the DNA bound by the protein. The merits of using the indirect measurement of ATP hydrolysis as a measure of DNA binding have been vetted for the *E*. *coli* RecA protein in numerous studies [71, 85, 87, 126, 128, 134–138], although an exception to the rule has been found recently for the RecA protein from *Deinococcus radiodurans* [114].

SSB inhibits the nucleation of RecA filament formation on ssDNA when it is present prior to RecA, leading to a substantial lag in binding as reflected in the DNA-dependent ATPase activity [87, 139]. Fig 2B shows a lag period of 28.6 ± 0.5 min for wild type RecA protein and much shorter lags of 12.8 ± 1.1, 4.7 ± 1.1 and 12.1 ± 1.9 min for I102L, V79L and E86G/C90G mutant RecA proteins, respectively. The final rates of ATP hydrolysis after SSB protein addition were observed and converted to a percentage of the maximum rate observed when RecA is bound prior to SSB addition. The wild type RecA protein attained 48.8% of its maximum rate, whereas RecA V79L, I102L and E86G/C90G reached higher rates of 74.6%, 98.6% and 80.9% of the maximum under these conditions, respectively. Displacement of SSB protein by the RecA protein variants appeared to be both faster and more complete than for wild type RecA protein. The results in Fig 2 represent the first of several indications that the RecA variants bind to DNA more persistently than the wild type protein.

#### The RecA variants exhibit only modest changes in several key RecA activities

For the wild type RecA protein, the exchange of RecA subunits between free and bound forms is limited when RecA filaments are formed on closed circular ssDNA and SSB is added after RecA. The exchange between free and bound forms increases substantially when DNA strand exchange is initiated [42, 43, 114, 140]. For the RecA variant proteins, these properties exhibited only a modest reduction in filament dynamics during DNA strand exchange (S6 Fig). We monitored a standard DNA strand exchange between circular ssDNA and homologous linear dsDNAs [36, 70] as promoted by the various RecA variants (S7 Fig). Reaction intermediates were produced at higher levels with the RecA variant proteins, particularly RecA I102L. However, those intermediates were converted to final products more slowly than was the case with the wild type protein. The results suggest a reduction in the observed coupling between ATP hydrolysis and DNA strand exchange [43, 126, 128, 141, 142] in the variants. Overall, the capacity of the RecA variants to promote DNA strand exchange was altered only slightly. DNA pairing by the RecA variants was also assessed directly, using a D-loop forming reaction assay (S8 Fig). Again, the RecA variants showed wild type levels of activity in most cases, although the RecA V79L mutant exhibited a higher capacity to promote D-loop formation at higher pHs (S8 Fig).

#### RecA variants are more resistant to the inhibitory effects of RecX protein

**Fig 3:**
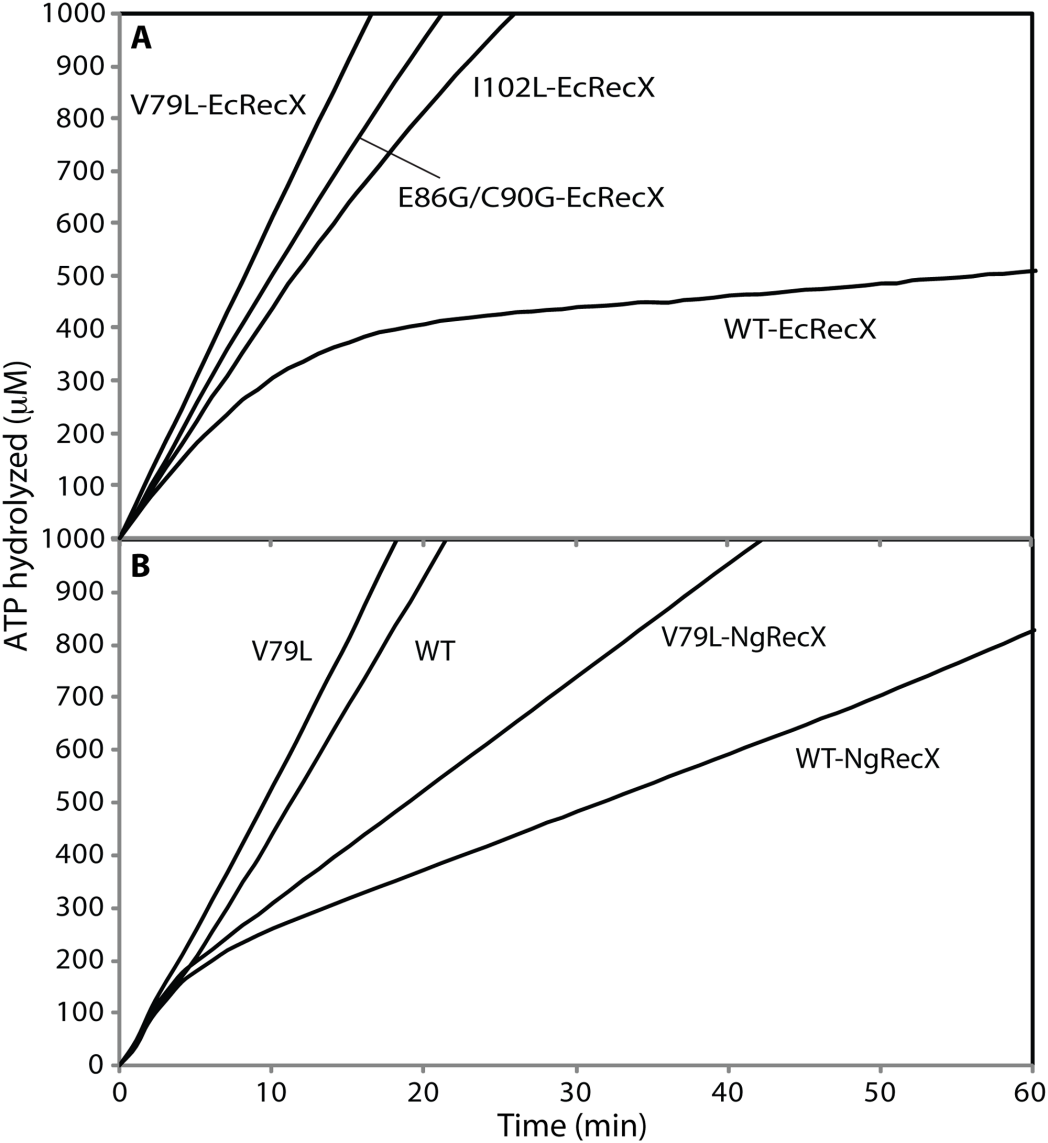
The effect of RecX proteins on ATPase activity of RecA variant proteins. (A) Effects of EcRecX protein. Reactions contained 3 μM RecA variant, 5 μM M13mp18 ssDNA, 0.5 μM SSB, and 3 mM ATP. The ATP hydrolysis by RecA protein was initiated by the addition of ATP and SSB protein. After 7 min, 100 nM of RecX protein was added and hydrolysis reaction was monitored as shown above. (B) The effect of NgRecX protein on ATPase activity of WT RecA and RecA V79L variant proteins. Reactions were carried out as in panel A, with NgRecX substituted for EcRecX protein where noted.

The RecX regulator protein and the UvrD helicase both play cellular roles in removing RecA protein from the DNA when it is no longer needed, as described in the Introduction. As the RecX protein plays a role in ameliorating the effects of RecA overexpression [101–104], we tested the RecA variants for sensitivity to this RecA regulator. The role of RecX becomes important in section 3 below. We first used the RecA-mediated ATPase activity to indirectly monitor RecA binding to ssDNA, and challenged reactions with EcRecX protein (Fig 3A). Addition of 100 nM of EcRecX protein after wild type RecA protein filaments had been formed resulted in an almost complete suppression of ATP hydrolysis that occurred within 20 min. This suggested that little cssDNA was left coated with wild type RecA protein because of the RecX protein binding, which blocked filament extension and caused net disassembly of the RecA filament. This pattern is consistent with a previous study in which the effect of RecX protein saturated as the RecX concentration approached 80–100 nM [88]. The RecA variants were much less sensitive to the EcRecX challenge. After the same 100 nM RecX protein addition, the rates of ATP hydrolysis by the three RecA variants, RecA V79L, E86G/C90G, and I102L, were 58.2 ± 1.4, 43.0 ± 3.1, and 32.7 ± 1.5 μM/min, respectively. These rates represented declines of between 20 to 45% relative to rates without RecX addition. Considerably more of the RecA variant proteins remained bound to cssDNA after RecX protein treatment. The least sensitive of the RecA variants, RecA V79L, was further tested for sensitivity to the more active RecX protein from *Neisseria gonorrhoeae*, NgRecX (Fig 3B). After addition of NgRecX, the wild type RecA protein settled into a lower rate of ATP hydrolysis. Importantly, the change occurred over a much shorter time span than is seen with an EcRecX challenge (compare with Fig 3A). For RecA V79L, a challenge with NgRecX also resulted in a rapid shift to a lower rate of ATP hydrolysis, indicating that the NgRecX has a greater effect on RecA V79L filaments than does the EcRecX protein.

**Fig 4:**
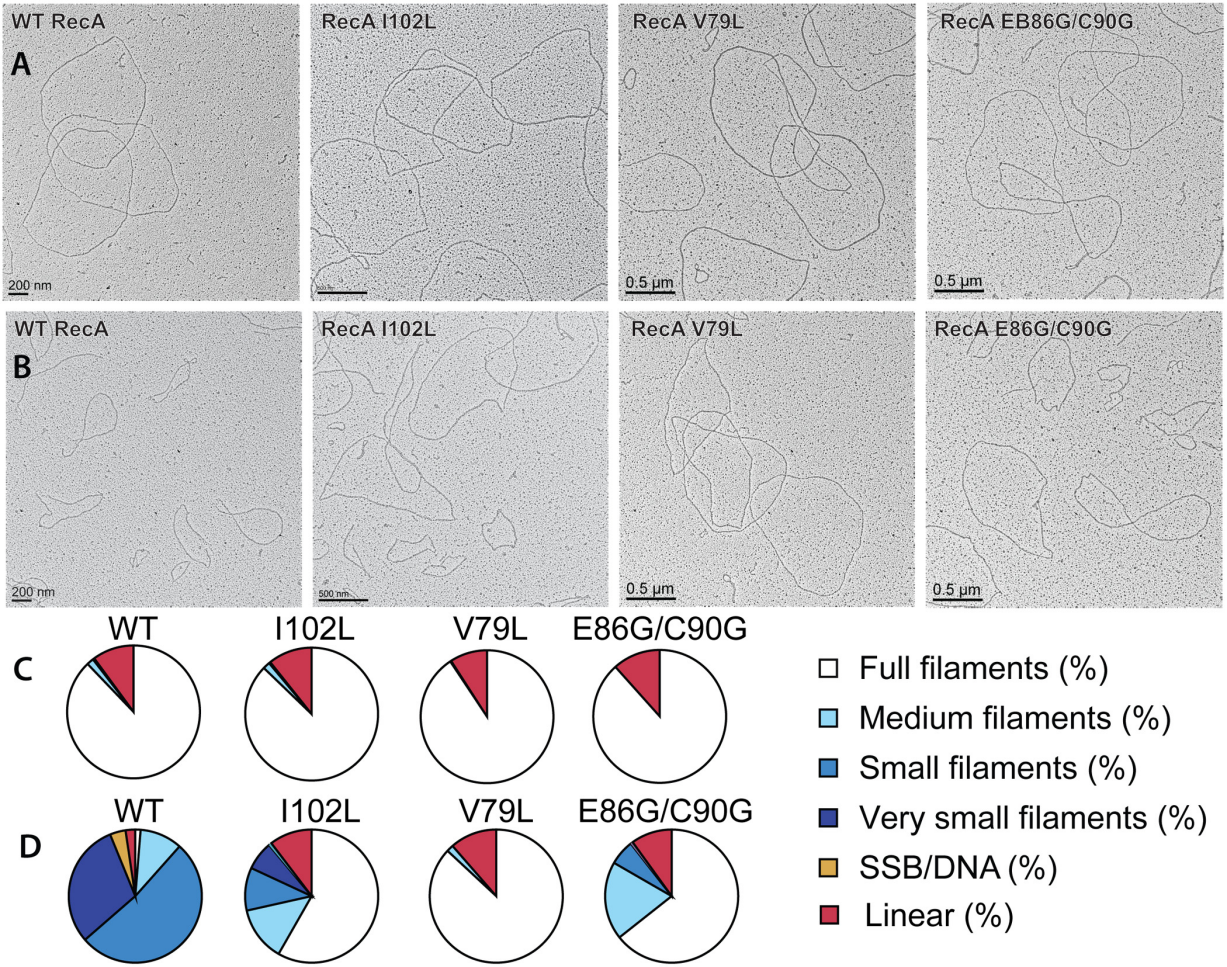
Electron microscopy of RecA variant protein filaments on cssDNA, with and without treatment by RecX protein. Electron micrographs show filament formation of wild type RecA and RecA variant proteins on M13mp18 ssDNA (A) without RecX protein and (B) with 100 nM RecX protein. RecA filaments were placed into 5 different categories based upon the size and completeness of filaments. (C) The composition of filaments in the various categories without RecX protein and (D) with RecX protein.

The reduced sensitivity of the RecA variants to EcRecX was confirmed by electron microscopy (Fig 4, Tables 1 and 2). RecA filaments were formed on M13mp18 ssDNA with or without RecX protein. When there is no RecX protein added, more than 87% of the observed molecules were full filaments in the reactions with wild type RecA protein as well as the RecA variants (Fig 4A and 4C). However, when 100 nM RecX protein was added to the reactions, the effect on the wild type RecA filaments was dramatic, whereas the effect on the RecA variant filaments was significantly reduced (Fig 4B and 4D). In the reaction of wild type RecA protein with RecX treatment, only 1.3% of the total molecules remained as full filaments, 10.3% were medium size filaments, 52.0% were small filaments, and 30.2% were very small filaments with large gaps. Approximately 3.8% of the DNA molecules had only SSB bound. In contrast, 58.3% of the molecules remained as full filaments after the RecA I102L mutant protein was treated with RecX. The remaining molecules had shorter RecA I102L filaments with very few SSB bound DNA molecules. The effect of RecX protein on RecA V79L filaments was particularly limited, as 86.9% of total molecules remained as full filaments after RecX treatment. The effect of RecX protein on RecA E86G/C90G variant filaments was slightly less than that seen with RecA I102L. The linear filaments (broken circles) were commonly observed about 9 to 11% in all experiments except the case of wild type RecA with RecX addition (2.4%) (Table 1). The average length of full, medium, small and very small filaments were measured from 10 randomly selected molecules and summarized in Table 2.

**Table 1:** Effect of EcRecX on RecA and RecA variant filaments.

	Total molecules counted	Full filaments (%)	Medium filaments (%)	Small filaments (%)	Very small filaments (%)	SSB/DNA (%)	Linear (%)
WT	1382	87.8	1.7	0.4	0	0	10.1
WT + EcRecX	3653	1.3	10.3	52.0	30.2	3.8	2.4
I102L	1458	87.2	1.7	0.4	0	0	10.7
I102L + EcRecX	1626	58.3	13.2	10.4	7.0	0.7	10.4
V79L	1147	90.6	0.2	0	0	0	9.1
V79L + EcRecX	1056	86.9	1.7	0	0	0	11.4
E86G/C90G	1125	88.4	0	0	0	0	11.6
E86G/C90G + EcRecX	1157	64.4	19.0	6.1	0.6	0	10.0

Quantitation of electron microscopy results. The fraction of observed molecules in each category described in the text and in Fig 4 are summarized.

**Table 2:** Measurements of RecA filaments of different size categories.

	Full filaments	Medium filaments	Small filaments	Very small filaments	SSB/DNA
WT RecA	3.72±0.07	–	–	–	–
WT + EcRecX	–	2.33±0.21	1.46±0.23	0.34±0.16	–
RecA I102L	3.70±0.09	–	–	–	–
I102L + EcRecX	3.70±0.08	2.35±0.31	1.12±0.26	0.49±0.23	–
RecA V79L	3.71±0.09	–	–	–	–
V79L + EcRecX	3.71±0.13	–	–	–	–
RecA E86G/C90G	3.63±0.09	–	–	–	–
E86G/C90G + EcRecX	3.75±0.10	2.49±0.21	1.51±0.39	–	–

Measurements of RecA filament lengths for each category were carried out as described in Materials and Methods. Lengths are reported in μm.

The DNA strand exchange activities of the RecA variants were also examined after a challenge with 50 nM RecX protein delivered at 7 min of reaction (S9 Fig). Here, both the wild type and variant RecA proteins exhibited strong reductions in activity, although the variants did produce somewhat higher levels of strand exchange products. To determine the extent to which the reduction in RecX inhibition affects the improvement in conjugational recombination exhibited by the RecA variants, a null *recX* (Δ*recX*) mutant strain was created and tested as a recipient in the previously established conjugational recombination assay. In the experiment with a plasmid encoding the wild type RecA protein, a deletion of the *recX* gene (EAW537) did not produce a significant change as shown in S10 Fig. This suggests that the RecX protein is not a limiting factor in conjugation promoted by the wild type RecA protein. However, when the plasmid expressed RecA V79L (EAW542), eliminating RecX increased the production of transconjugants slightly (~1.3 fold) using the reaction protocol requiring 4 crossovers. When the RecA V79L mutant protein was expressed from the recipient chromosome, 3.7 and 5.0 times more transconjugants were produced than wild type RecA protein in the presence of wild type *recX* and in the Δ*recX* context, respectively. Thus, in the cell, the gains in conjugational recombination seen with the RecA V79L variant are actually limited somewhat by the EcRecX protein, in spite of its relatively modest effects on the RecA V79L protein.

### RecA variants with enhanced conjugation function are barriers to other processes in DNA metabolism

#### Enhancement of RecA-mediated conjugational recombination does not correlate with positive or negative effects on other RecA cellular functions

Why has nature not produced further improvements in RecA recombination function via evolution? We wished to determine if the *recA* mutations that conferred a conjugational enhancement had deleterious effects on the cell. We first tested the UV radiation and ciprofloxacin sensitivity of mutant strains expressing the RecA variants in order to determine if the enhancements in conjugational recombination output were mirrored in other cellular functions requiring RecA protein. In all cases, the RecA variant proteins were expressed on the chromosome at the normal *recA* locus.

**Fig 5:**
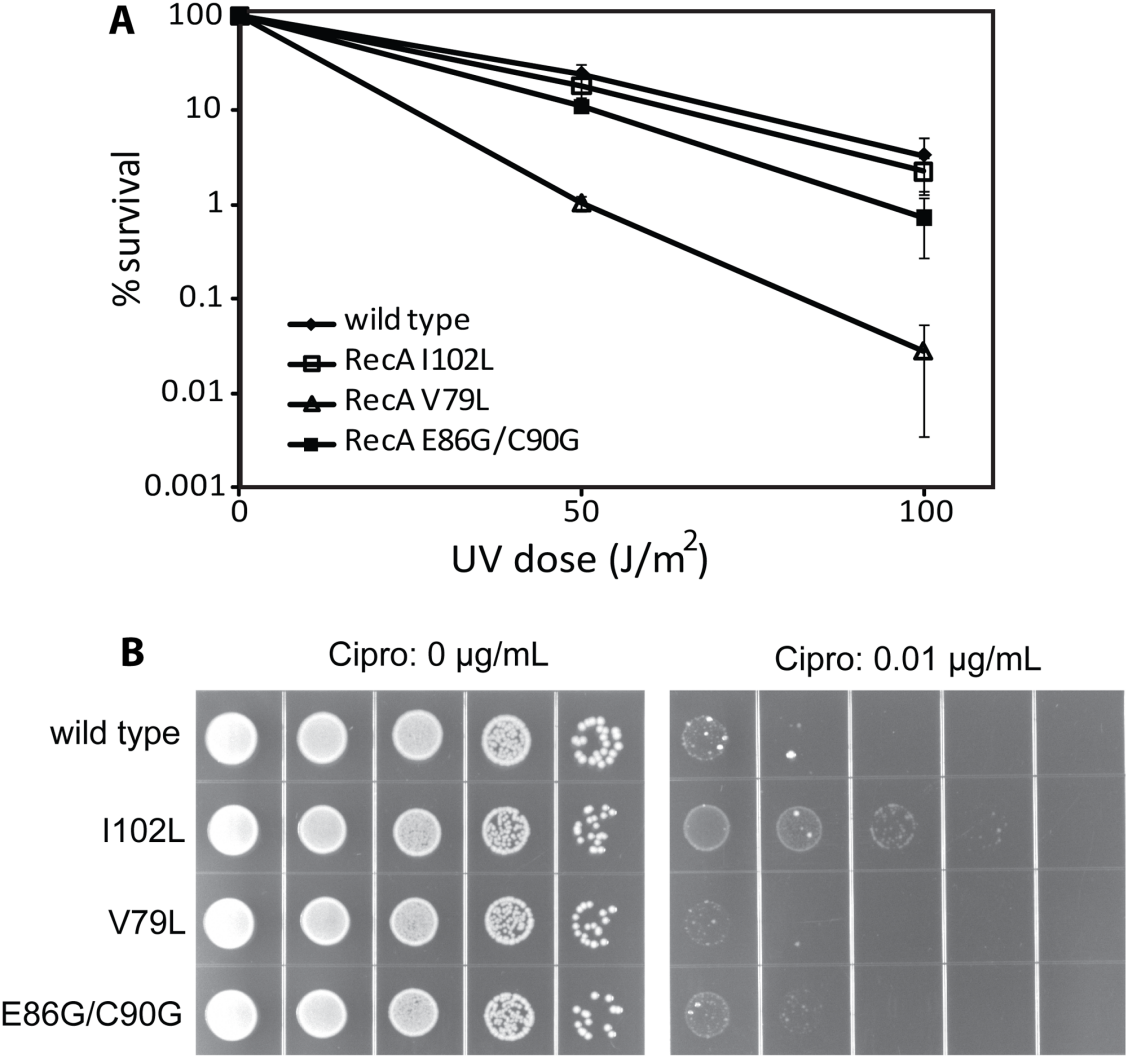
The effects of RecA variants on UV radiation and ciprofloxacin sensitivity. (A) Mutant strains expressing wild type RecA or the indicated RecA variant proteins were plated and exposed to UV radiation as described in Materials and Methods. The colonies were counted to obtain viability data, which was normalized against the zero dose point to obtain percent survival. (B) Cells were grown to log phase, serially diluted 1:10, and spot plated on LB plates either with or without 0.01 μg/ml ciprofloxacin as indicated.

As shown in Fig 5A, some of the mutant strains exhibited defects in viability relative to an isogenic wild type *recA* strain (EAW 105) after UV exposure. The mutant strain expressing RecA V79L (EAW 394) was most sensitive to UV radiation after both the 50 and 100 J/m^2^ doses. The other mutant strains, EAW 334 and EAW 410, expressing RecA I102L and E86G/C90G, respectively, showed a less severe effect than the EAW 394. We conclude that an enhancement in conjugational function is not carried over into other RecA functions in the cell, and in fact can be deleterious to the normal DNA repair functions of RecA protein in at least some cases.

To further explore the effects of the RecA variants *in vivo*, we tested for sensitivity to the double strand break inducer ciprofloxacin. Ciprofloxacin is an inhibitor of gyrase that traps covalent protein-DNA adducts resulting in double-strand breaks during replication, transcription, or proteolysis. In this experiment, quantitative analysis was not available since colonies grown on ciprofloxacin plates were of widely varying sizes, making colony counting impractical. However, dot pictures employing serial dilutions were clear enough to show differences in survival conferred by the wild type RecA protein and variants. As shown in Fig 5B, wild type RecA exhibited a serious growth defect at 0.01 μg/ml ciprofloxacin concentration. Expression of the RecA I102L appeared to increase viability somewhat. For the other two variants the changes were negligible. Thus, an enhancement of conjugational recombination activity does not alter other aspects of RecA in vivo function in predictable ways. The aspects of RecA function that are most important in conjugational recombination are not the same as those most critical to DNA repair and other RecA-dependent processes.

#### Expression of the RecA variants produces a growth defect explained by more persistent DNA binding

In early trials, strains expressing the various RecA variants appeared to grow somewhat more slowly in culture, implying a modest growth deficiency that is further explored here. As already noted, cellular toxicity could in principle arise due to extreme levels of genetic exchange. In the absence of a large increase in a key recombination intermediate (single stranded DNA gaps or ends), it seemed unlikely that the RecA variants would generate levels of genetic exchange sufficient to slow cell growth. In addition, the *in vitro* work revealed no systematic and substantial increases in the DNA pairing and strand exchange functions of RecA that might elevate genetic exchange levels sufficiently to account for a growth deficiency. We thus focused on two additional explanations for the growth deficiency: a potentially deleterious constitutive expression of the SOS response or the creation of barriers to replication and/or transcription by persistently bound RecA filaments.

**Fig 6:**
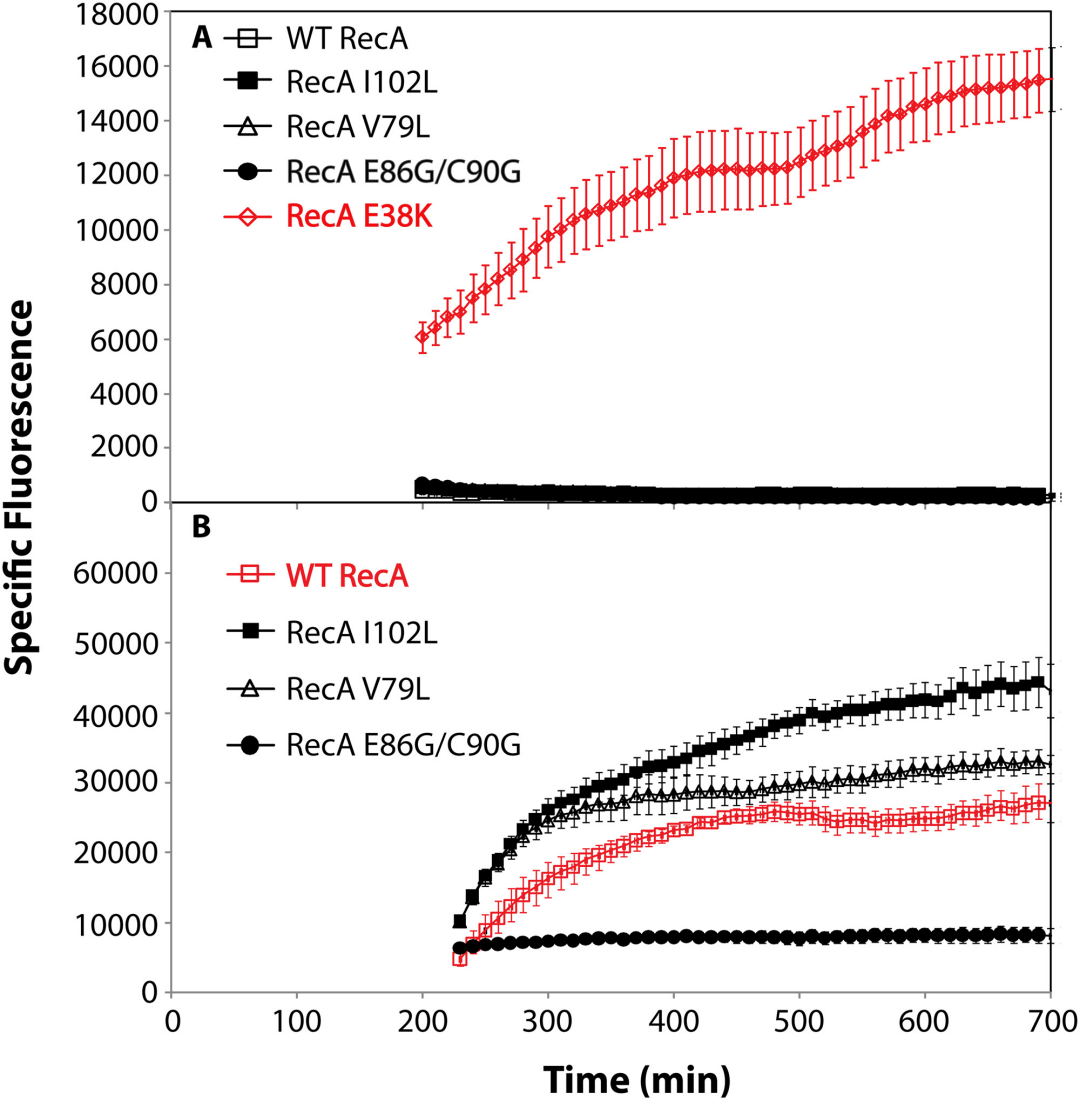
SOS response of RecA variant proteins. RecA variant strains containing a plasmid expressing GFP under SOS control (utilizing the *recN* promoter) were grown in LB. Specific fluorescence, defined as measured fluorescence divided by the OD_600_, is shown. (A) The SOS response in cells expressing RecA variant proteins without treatment with any DNA damaging agent. (B) The SOS response in cells expressing RecA variant proteins after induction by adding 0.005 μg/ml ciprofloxacin at 180 minutes. Due to the error inherent in dividing very small numbers, specific fluorescence is not displayed for times prior to 200 and 230 min in panels A and B, respectively.

The induction of the SOS response is examined in Fig 6. GFP expression from the SOS *recN* promoter was used to monitor this function. None of the RecA variants produced a constitutive SOS response that rose above levels normally seen in wild type cells under normal growth conditions (Fig 6A). The RecA E38K mutant (RecA 730), in which the SOS response is demonstrably constitutive [143–146], was included in the experiment as a positive control. All of the RecA variants displayed a capacity to induce the SOS response when the cells were challenged by addition of ciprofloxacin (Fig 6B), although the levels achieved by the E86G/C90G variant were substantially reduced relative to the other two variants and the wild type protein.

**Fig 7:**
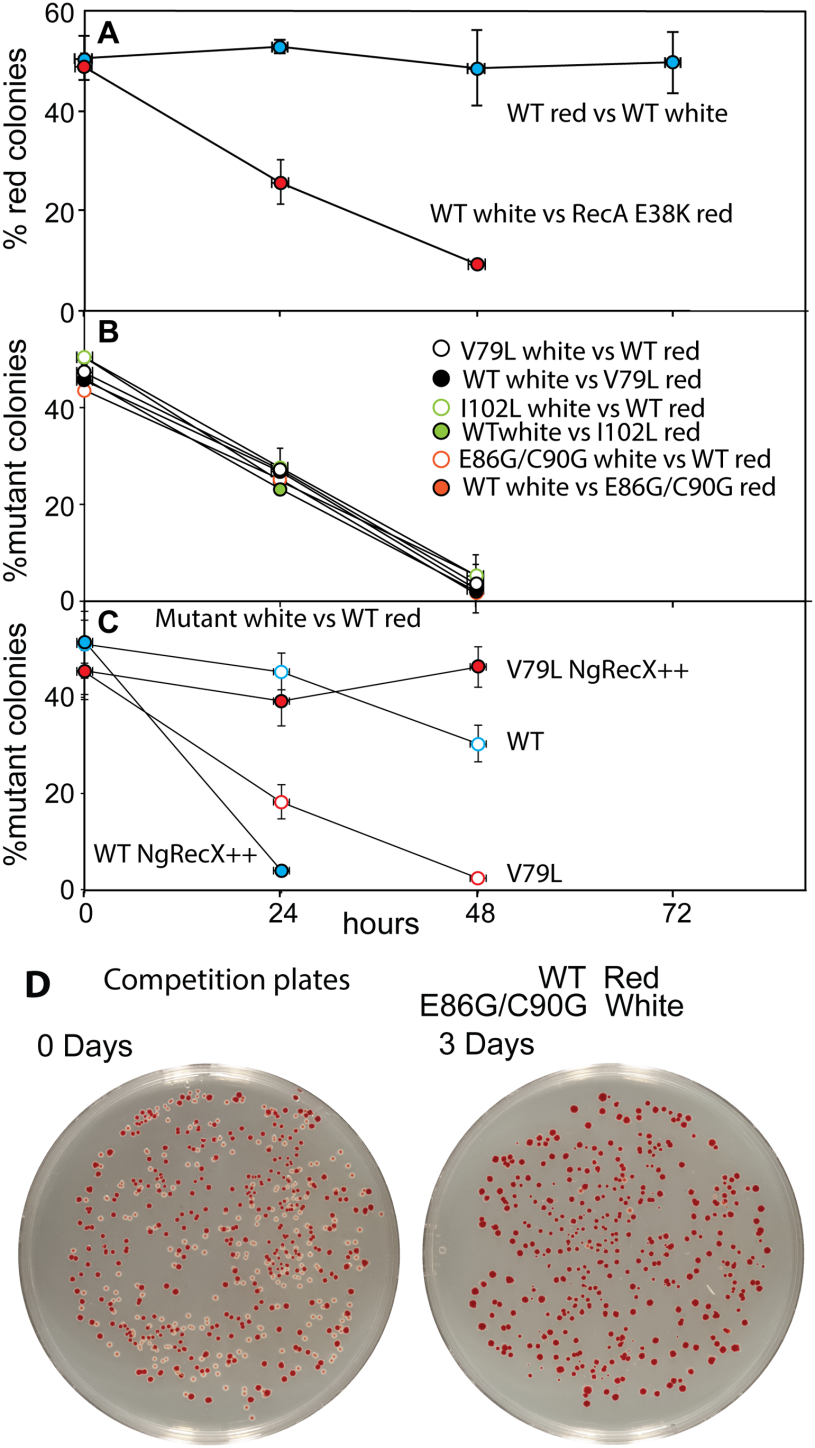
Cell growth competition assays. Assays were carried out as described in Materials and Methods. (A) Two trial competitions. The top trial shows a competition between two wild type cultures, one of which carries the Ara^–^ mutation. The lower one shows a competition between wild type cells and cells expressing RecA E38K. As is the case for the RecA variants studied here, RecA E38K also confers a growth disadvantage on cells in which it is expressed. Colony counts revealing the % of cells expressing the mutant RecA proteins with enhanced conjugational function are plotted as a function of the daily growth cycle of the experiment. (B) Competitions between cells expressing each of the three variant proteins and wild type cells. Two competitions are shown for each, with the Ara^–^ mutation either in the mutant or wild type cells. (C) Competitions between wild type cells (red) and cells with a gene expressing NgRecX protein from the normal *recX* locus on the *E*. *coli* chromosome. The cells expressed either the wild type RecA or RecA V79L from the *recA* locus as indicated. In two cases, a plasmid also expressing the NgRecX protein at higher levels (pEAW947) was included (NgRecX++). (D) Examples of competition plates from an earlier trial, showing the mixtures of red and white colonies before and after the three days of growth cycles.

We then examined the possibility that the RecA variants simply bound too tightly to the DNA, generating a barrier to replication and other aspects of DNA metabolism. To provide a more sensitive measurement of any deleterious effects of the *recA* mutations on cell growth and survival, we carried out direct competition assays between strains expressing wild type and mutant RecA proteins all present at the same normal chromosomal locus [147] (Fig 7). Wild type or mutant cells were modified to carry a neutral Ara^–^ mutation (which confers a red color on colonies when grown on tetrazolium arabinose (TA) indicator plates) to permit color based scoring of mixed populations [147]. Overnight cultures of cells expressing one of the three *recA* variants most thoroughly characterized above were mixed in a 50/50 ratio with isogenic wild type cells carrying the Ara^–^ mutation, or vice versa. A sample of each mixture was diluted 10^−6^ and plated on TA indicator plates to generate approximately 200 colonies. The cell mixtures were diluted 1/100 into fresh L broth and grown overnight. Plating, dilution, and overnight growth were repeated two more times, and each of the four experiments was done in triplicate. Red and white colonies were counted for each plating, and the percentage of cells expressing the mutant RecA protein was determined for each successive plating.

As shown in Fig 7A and in earlier work [116, 147], the Ara^–^ mutation does not affect growth rates by itself. Ratios of red (Ara^–^) and white cells remained in an approximate 50/50 ratio for three days after the experiment was initiated. We also tested the RecA E38K mutant that displaces SSB rapidly and induces the SOS constitutively (see Fig 6A) to determine its effects on cell growth. Cells expressing RecA E38K on the chromosome in place of the WT RecA protein were eliminated from the competition after two days of growth.

In all competition experiments, cells expressing the wild type RecA protein rapidly displaced the cells expressing each of the three RecA variant proteins (each expressed at the normal *recA* locus on the chromosome), such that few cells with the mutant proteins remained after 2–3 cycles of overnight competitive growth (Fig 7B). The results were identical whether the Ara^–^ mutation was present in the mutant or WT cells within the competition.

We carried out an additional experiment with the V79L variant to determine the cause of the growth deficiency (Fig 7C). As already noted, the effects of EcRecX on the RecA V79L protein are relatively modest. However, we wished to determine if the more robust NgRecX could alleviate the growth deficiency. In this experiment, all of the “white” strains had NgRecX replacing the EcRecX on the chromosome, and are placed in competition with wild type “red” cells. As shown in Fig 7C (open symbols), the presence of the NgRecX expressed from the chromosome had a modest deleterious effect on cells expressing the WT RecA protein, and did not alleviate the growth deficiency seen in cells expressing RecA V79L. However, when a plasmid was introduced that expressed the NgRecX at higher levels, the situation changed. Cells with the wild type RecA protein declined precipitously due to toxic effects of the NgRecX protein. Given the strong effects of NgRecX on the EcRecA protein [90], cells expressing high levels of NgRecX (as here) may be effectively *recA* ^–^. In contrast, the growth deficiency of cells expressing RecA V79L was almost entirely alleviated. The separate negative effects of expression of RecA V79L and high levels of NgRecX come together here to create a new balance that permits the cells to grow at normal rates. An example of plates before and after the growth cycles of one competition is provided in Fig 7D.

We conclude that expression of the RecA variant proteins, even from the normal *recA* locus, confers a significant growth disadvantage. In at least the case of RecA V79L, the growth deficiency appears to be caused by an overly persistent binding of the RecA variant to DNA, leading to a presumed barrier to other aspects of DNA metabolism. Forced disassembly of the RecA filaments by the robust NgRecX protein is detrimental to the wild type RecA protein, but is sufficient to bring DNA binding and filament disassembly back into balance for the RecA V79L variant. The positive effect of high levels of NgRecX implies that cell growth rates can be restored by breaking up DNA-bound filaments of the RecA V79L protein.

## Discussion

There are three main conclusions to this work. First, the wild type *E*. *coli* RecA protein has not evolved to optimize the genetic exchanges required for conjugational recombination. Substantial increases in recombinase function can be obtained. Second, the observed functional improvements in conjugational recombination may involve many, sometimes subtle changes in protein activity. In this study, not all the changes are subtle. The one feature found in common for the three RecA variants arising most prominently in this study is a more persistent binding to DNA. This is reflected in substantially more rapid displacement of SSB for nucleation onto ssDNA (Fig 2), and a greatly reduced sensitivity to the RecX inhibitor protein (Figs 3 and 4). Third, the improvements in conjugation function come only at the cost of a growth deficiency evident for all three RecA variants in competition experiments. For RecA V79L, that growth deficiency reflects the increased DNA binding persistence. Normal growth is restored by overexpression of the more robust RecX protein from *Neisseria gonorrhoeae* (Fig 7). The growth deficiencies displayed by cells expressing the other two RecA variants might be explained by a similar mechanism. The work reveals a critical evolutionary compromise between necessary DNA repair processes and potentially deleterious genomic effects.

We previously noted the existence of RecA mutant proteins with enhanced recombination potential [118]. In the current study, we have used a selection to generate variants with this same capacity for greater recombination. The selection protocol is robust and reproducible. The improved function of these RecA variants may provide a more robust platform for the continued investigation of key recombinase activities. The increases in recombination documented in this study are reflected in many changes in RecA protein activities, but many of them are subtle and unlikely to account for the enhancement on their own. ATP hydrolytic rates are increased, but the coupling between ATP hydrolysis and DNA strand exchange appears to be reduced (DNA strand exchange actually proceeds slower rather than faster). DNA pairing is improved for some of the RecA variants, but this is evident only at high pH. Our working hypothesis is that the enhanced conjugational recombination reflects an overall increase in RecA filament persistence on the DNA. This is seen in multiple assays in which the rates of filament nucleation on SSB-coated ssDNA are increased for the RecA variants, and the rates of RecA filament disassembly (in the presence of RecX or RecA K72R) are decreased. Since ATP hydrolysis rates increase, a reduction in filament disassembly must come about via a decreased coupling between ATP hydrolysis and RecA subunit dissociation at the 5'-proximal end. That persistence in binding is perhaps best encapsulated by the greater overall binding of the RecA protein variants to short oligonucleotide DNA substrates.

To promote conjugational recombination, RecA protein must bind to the transferred single stranded DNA and carry out a complete genomic search for homology. In this context, more persistent binding by a RecA filament makes sense. Improvements in this parameter should increase the length of time available for a homology search and improve chances that a productive pairing will occur. In the context of recombinational DNA repair at a replication fork, persistent binding of a RecA filament to DNA is not necessary and probably detrimental. At a replication fork, the homologous DNAs to be paired are generally in close proximity; a widespread genomic search for homology is not needed. A RecA filament that overstays its welcome will simply be a barrier to productive replication restart.

A substantial reduction in sensitivity to the inhibitory EcRecX protein, seen for all three characterized RecA variants, makes a significant contribution to the overall DNA binding persistence that would occur in the cell. Elimination of *recX* function does not have major phenotypic consequences in wild type *E*. *coli* cells [105]. RecX helps to maintain an optimal balance between active (bound) and disassembled RecA protein in the cell. A decline in sensitivity to EcRecX helps lead to growth deficiencies, and expression of a more robust version of RecX protein can restore balance. The evolutionary significance of RecX is thus rendered more apparent. In addition to RecX, the UvrD helicase has a major role in removing RecA filaments from the DNA to keep them from impeding other aspects of DNA metabolism [98–100, 148, 149]. Recent work has shown that UvrD is defective in displacing RecA variants with enhanced DNA binding properties such as RecA E38K [100]. This might also help explain the observed growth deficiencies in strains expressing our RecA variants.

The current work begins to build a case that RecA filaments can represent substantial barriers to replication and possibly to transcription, and that those barriers have cellular consequences. An earlier and extreme example of RecA as a barrier came in the form of the RecA K250R mutant, which hydrolyzes ATP six times more slowly than the wild type protein [126]. This leads to a six-fold decrease in rates of filament dissociation from DNA, and an accompanying six-fold decline in cell growth rate [126]. Suppressors arise quickly in strains expressing RecA K250R, most of them inactivating the mutant *recA* gene [126]. Collisions between replication forks and bound recombinase filaments could have genome instability implications in all cells.

The mutagenesis and selection method used here focused on one region of the protein representing about 17% of the amino acid residues in RecA. Within this region, we have queried every possible single base substitution with 90% confidence, and the library included some double, triple, and quadruple mutant proteins (the library did not cover nearly all the possible combinations of multiple mutations). The region selected, between residues 79 and 137, is not the only part of the protein with the potential to generate variants with increased recombination potential. It was selected due to the presence of changes in the region that were previously shown to produce the desired phenotype. A complete assessment of changes that could affect RecA function in this way will require screens focusing on other *recA* gene segments.

RecA was originally discovered due to its effects on conjugational recombination [21], and many early studies of *recA* were carried out in this context. The lack of optimization for conjugation during evolution, coupled to the growth deficiency that accompanies enhancement of this process, provides yet another argument that recombinases did not evolve to promote chromosomal genetic exchanges per se [11–14, 16, 126, 150]. Instead, recombination evolved to repair double strand breaks [11–14, 16, 126, 150]. Genetic exchanges during conjugation, and perhaps eukaryotic meiosis, reflect an evolutionary repurposing of pre-existing systems. The functional compromise between the positive and negative effects of recombinases and recombination seems likely to take different forms in different species.

**Fig 8:**
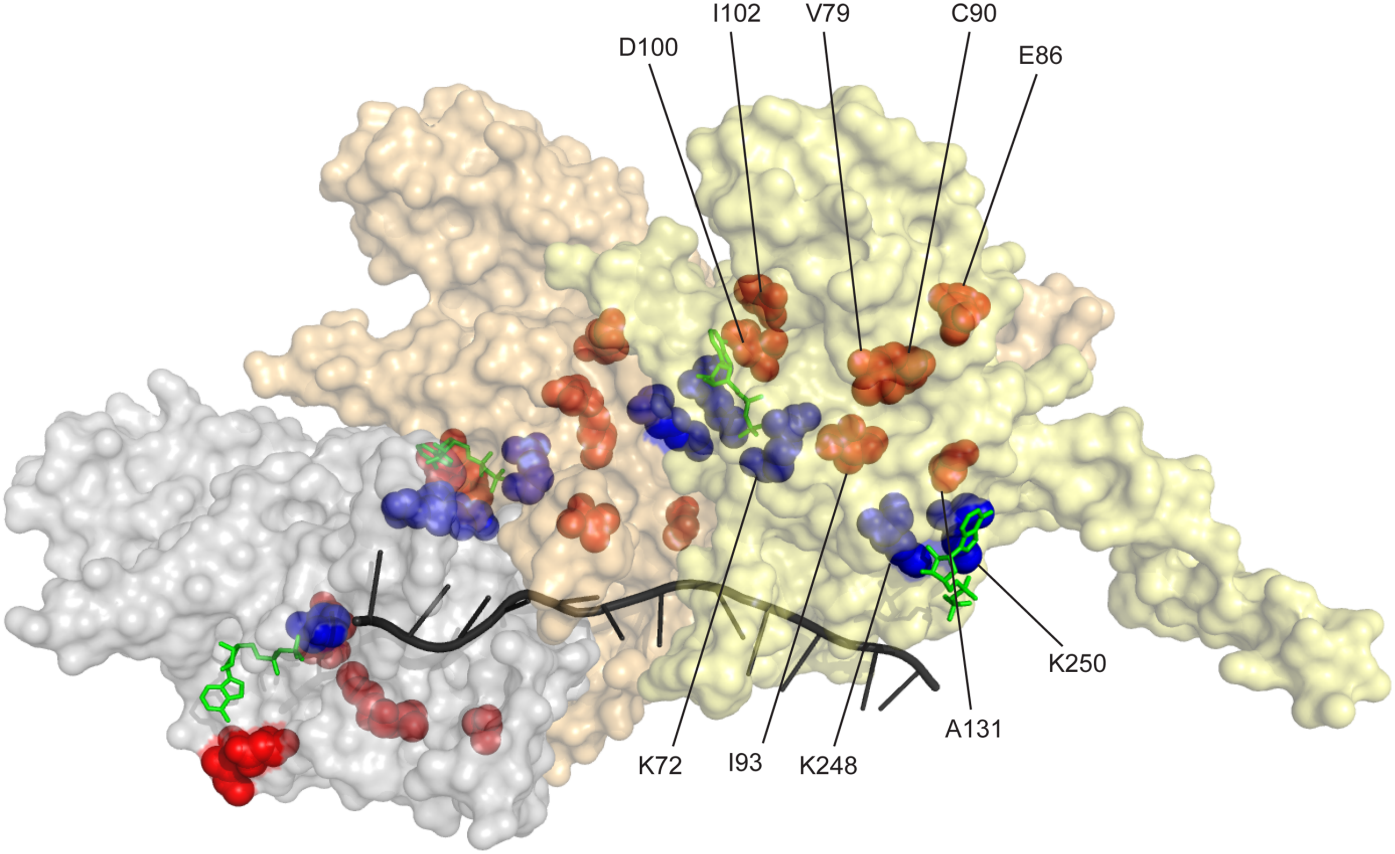
RecA protein amino acid residues affected in RecA variants with increased recombination potential. Three RecA subunits in a RecA-ssDNA nucleoprotein filament (from coordinates provided by Pavletich and coworkers [165]) are shown, with a surface contour rendering in which each subunit is transparent but differently colored. The path of the ssDNA within the filament is shown by the black helical line. ADP residues are shown in green. The ATPase active site is at the subunit-subunit interface. Three residues at the ATPase active site (K72 on one face and K248 and K250 on the opposing face) are shown in blue. Prominent residues in which amino acid changes bring about enhanced recombination potential are shown in red/orange.

The residues affected by the more prominent mutations identified in our two separate selective screens are highlighted in Fig 8 (orange/red). Key residues bracketing the ATPase active site at the subunit-subunit interface (K72 on one side [151, 152] and K248 and K250 on the other [126, 136]) are shown in blue. Some of the residues identified in this study are at the subunit-subunit interface (D100, D102, E86, C90, A131), but others are not (V79, I93). We hypothesize that the variants in all of these residues may affect coupling of ATP hydrolytic events to conformational changes and/or general allosteric communication between subunits. This communication may in turn affect rates of filament disassembly. Continued work should elucidate subtle structure-function relationships that affect all aspects of the coupling of ATP hydrolysis to RecA function. The wild type RecA protein of *Escherichia coli* seems to have evolved to do its job quickly and get out of the way.

## Materials and Methods

### DNA substrates and strains

Supercoiled double-stranded DNA and circular single-stranded DNA from M13mp18 bacteriophage were prepared as described previously [153]. Linear double-stranded DNA for strand exchange reactions was generated by complete digestion of supercoiled DNA with PstI restriction endonucleases. For D-loop forming reaction assays, 8 units of T7 exonucleases per μg of DNA were used for an additional digestion of double-stranded DNA to form 150 nt long 3' overhang. The concentration of dsDNA and ssDNA substrates were determined using absorbance at 260 nm and the conversion factors 108 μM A260^-1^ and 151 μM A260^-1^, respectively. DNA concentrations are expressed in terms of total nucleotides.

Donor EAW175 and recipient EAW188 strains were constructed by P1 transductions from several strains. EAW175 was made by a consecutive P1 transduction of, first, the Δ (*metA*)::*kan* llele from SS6311 into CAG5052 (KL227 *btuB3191*::Tn*10 metB1 relA1* 89′→6′) to obtain an intermediate strain EAW173, checked by Tet^r^ and Kan^r^ phenotypes, then followed by kan flipping out and, second, the *ilvO*::*kan* allele from SS4761 into EAW173 strain, checking for both Tet^r^ and Kan^r^ phenotypes.

To make recipient strain, kan was flipped out first from SS338 (Δ (*attB*)::*psulA*-*gfp* Δ (*metE*)*100*::*kan*) strain and intermediate strain EAW174 was made by P1 transduction of the Δ (*aroB*)::*kan* allele from SS2495 to SS3388. The Δ*recA*::*kan* allele from EAW20 was then transferred to EAW174 by P1 transduction to make recipient EAW188. EAW188 was transformed with pT7POL26.

EAW334 = MG1655 with recA I102L on the chromosome in the recA locus EAW334 was constructed using a variation of the procedure of Datsenko and Wanner [154]. A plasmid with the MG1655 region from the 200 bases upstream of the *recA* gene to 210bp downstream of the stop of the *recX* gene was constructed. A cassette containing the KanR gene flanked by a mutant FRT and a wt FRT was added just downstream of the stop of the *recX* gene to use as a removable marker. This plasmid was designated pEAW675. A plasmid containing the recA gene with an I102L mutation was digested with NcoI and EcoRI and the mutant DNA fragment was ligated into pEAW675 digested with the same enzymes. The plasmid, designated pEAW884 was directly sequenced to confirm the presence of recA I102L. pEAW884 was used as a template in a PCR with primers consisting of bases 200–180 before the start of *recA*, and 210–192 after the end of recX. The PCR product was electroporated into EAW20, which is MG1655Δ *recA*, containing the plasmid pKD46. A Kanamycin resistant colony was used as template in a PCR, and the product was sequenced to confirm the presence of *recA* I102L. The KanR cassette was popped out by transforming the strain with the FLP expression plasmid pLH29, and incubating with IPTG.

EAW394 = MG1655 with recA V79L, and EAW410 = MG1655 with recA E86G+C90G on the chromosome in the recA locus. EAW394 and 410 were constructed in a manner similar to EAW334, with the plasmid containing recA V79L, or E86G/C90G digested with NcoI and EcoRI and ligated into pEAW675 digested with the same enzymes.

pEAW947 = Ng recX in pBAD/Myc-HisA. Plasmid Ng *recX* (Siefert Lab) was used as the template in a PCR with a primer consisting of a BspHI site followed by the bases 5–32 of the Ng *recX* gene. The BspHI site contains bases 1–4 of the start of the Ng *recX* gene. A change was made for better codon use at Leu7. The other primer consisted of a BamHI site followed by the last 24 bases of the Ng recX gene. The PCR product was digested with BspHI and BamHI and ligated to pBAD/Myc-HisA(Invitrogen) digested with NcoI and BglII, enzymes having compatible cohesive ends with BspHI and BamHI. The resulting plasmid, designated pEAW947 was directly sequenced to confirm the presence of Ng recX.

Construction of ΔaraBAD strains EAW214, 564, 568, 569. EAW214 was constructed using a variation of the procedure of Datsenko and Wanner [154]. pEAW507, a plasmid containing a mutant FRT-KanR- wt FRT cassette, was used as a template in a PCR. The primers consisted of the 51 bases before the start of the araBAD promoter +20 bases before the mutant FRT, and the 51bases after the stop of araD+21 bases after the other FRT. The PCR product was electroporated into MG1655/pKD46, and a Kanamycin resistant colony was selected. DNA from this colony, designated EAW214, was used as a template in a PCR to confirm the presence of the FRT-Kan R-FRT replacing the araBAD promoter and genes on the chromosome. P1 transduction was used to transfer the araBAD deletion into EAW394, 334, and 410. The resulting strains were designated EAW564, 568, and 569. DNA from these strains was used as templates in PCRs to confirm the presence of the FRT-Kan R-FRT replacing the araBAD promoter and genes on the chromosome.

EAW575, and 578 = Gc *recX* on the chromosome in the Ec recX locus of wt recA, and recA V79L. EAW575, and 578 were constructed using a variation of the procedure of Datsenko and Wanner [154]. The mutant FRT-KanR- wt FRT cassette from pEAW507 was excised by EcoRI and SalI digestion, and inserted after the end of the Gc *recX* gene of plasmid pEAW947, which was digested with the same enzymes. The resulting plasmid, designated pEAW1016, was used as template in a PCR with primers consisting of the 51bp of the *E*. *coli* chromosome before the start of *recX* +the first 21 bp of the Gc *recX* gene, and the 51bp of the *E*. *coli* chromosome after the end of *recX*+21 bases after the wt FRT of pEAW1016. The PCR product was electroporated into MG1655, and a kanamycin sensitive version of EAW394, both containing the plasmid pKD46. DNA from these strains was used as templates in PCRs, and sequenced to confirm the presence of wt recA + Gc *recX* for EAW575, and *recA* V79L+Gc *recX* for EAW578.

### Proteins

The *E*. *coli* RecX, *Neisseria gonorrhoeae* RecX [90], SSB [90] and the wild type RecA protein [155, 156] were purified as previously described. The RecA V79L, RecA I102L, RecA E86G/C90G mutant proteins were purified by the same means as the wild type RecA protein with the following modifications. The plasmids encoding the mutant *recA* genes were transformed into the Δ*recA* and nuclease-deficient strain STL2669. The Polyethylenimine pellet was washed with R Buffer (20 mM Tris-Cl buffer (80% cation, pH 7.5), 0.1 mM EDTA, 10% (w/v) glycerol, 1 mM dithiothreitol) and extracted twice with R Buffer plus 300 mM ammonium sulfate. After precipitation by ammonium sulfate to 50% saturation, the pellet was resuspended in R buffer plus 1 M ammonium sulfate. Proteins were purified using chromatography on some combination of Butyl-Sepharose, Ceramic HAP, Source 15S, Source 15Q, DEAE sepharose columns. Between columns, peak fractions were identified by SDS-PAGE and pooled together before dialyzing, if necessary. The concentrations of *E*. *coli* RecX, SSB and RecA proteins were determined from the absorbance at 280 nm using the native extinction coefficient 2.57 × 104 M^−1^ cm^−1^ [88], 2.38 × 104 M^−1^ cm^−1^ [157] and 2.23 × 104 M^−1^ cm^−1^ [158], respectively. The purified proteins were free of detectable nuclease activities on double stranded DNA and single stranded DNA.

### Library constructions

The oligonucleotide cassettes-directed method [159] from earlier study was modified to create randomized libraries for *E*. *coli* RecA protein. The cassette mutagenesis procedure involves the synthesis of a small, double-stranded DNA molecule that can be ligated into a larger vector fragment to reconstruct the gene of interest [160]. As an in vivo expression vector, the plasmid pACYC184 with T7 promoter and *recA* gene was digested with restriction enzymes SapI and PstI to generate a backbone fragment. A double-stranded DNA fragments corresponding to the region between sites SapI and PstI was made by annealing three separate oligonucleotides. Only two oligonucleotides were randomly mutated through incorporation of degenerate DNA sequence using different molar ratios of four nucleotides as mixtures during synthesis. The ratios were 99% to 0.33% and 98.5% to 0.5%, corresponding to wild-type base to each of the other bases. These two oligonucleotides were placed abreast and annealed to the other complementary oligonucleotide to make randomized double-stranded DNA molecule. The last oligonucleotide was synthesized without mutations to avoid too many mismatches, thus increase annealing efficiency between complementary strands. This small DNA inserts were then ligated to the backbone fragment to generate the mutagenized plasmid library, which was transformed into the DH5α cells by electroporation. More than 27,500 grown colony isolates were combined in a pool and the population of recA gene-bearing plasmid was purified. The recipient cells were transformed with the purified plasmid DNA pool for conjugational assay.

### Confidence intervals

In order to determine the number of clones necessary to achieve 90 and 95 percent confidence of the presence of all 531 clones, a Monte Carlo simulation was designed. A simple code was written, using Python (http://www.python.org), to pick a number out of 531 at random and keep picking numbers until the entire set of numbers 1 to 531 was selected. The total number of selections needed to complete the set was recorded for each trial, with the trial ending when the entire set of numbers was selected. With each number from 1 to 531 representing a different possible mutation, the total number of clones needed to get all 531 mutations for each trial was represented by this total.

One million trials were run using this code. A 90 percent confidence level that all 531 mutations are present in a group of clones means that more than 900,000 trials must have a number of clones less than the group in question. Similarly, to be 95 percent confident, 950,000 trials must have a number of clones less than the group in question. This was done in Excel by totaling up the histogram data from the simulation and finding the minimum number of clones needed to obtain the entire set of 531 mutations in at least 900,000 and 950,000 trials, respectively.

See the supplementary materials for the source code for this simulation.

### Conjugational recombination assays

Conjugation was carried out essentially as described before [161] with following exceptions. Donor strain was grown at 37°C in Luria-Bertani (LB) broth with Tetracycline until an optical density (OD_600_) of 0.7 was reached. Recipient strain was grown with Chloramphenicol, Kanamycin and Streptomycin until an optical density (OD_600_) of 0.4 was reached and then induced for 40 minutes with Isopropyl β-D-1-thiogalactopyranoside (IPTG) of final 40 μM. The concentration of IPTG was optimized for producing about 700–800 recombinant colonies per 1,000,000 donors at a cross requiring transfer of one marker (two crossovers), using recipients expressing wild type RecA protein from the same expression used in library construction. Both strains were spun down and gently resuspended in the amount of initial volume of fresh LB broth to remove antibiotics. Mating was carried out by mixing 200 μl of donor cells with 1800 μl of recipient cells and incubating 100 min at 37°C. The 200 μl of the mating mixture was mixed with 3 ml of pre-warmed 0.7% Bacto agarose solution to prevent additional mating and immediately poured onto a selective media plate. The plate was sat for a few minutes at a room temperature and turned upside down and incubated for 40 hours at 37°C.

During repeated rounds of conjugation, all of mating mixtures were poured onto selective media plates. After each round, the resulting recombinant colonies were combined in a pool and the population of *recA* gene-bearing plasmids was isolated and stored. The isolated plasmid pool was introduced into a new batch of *recA* ^-^ recipient cells for the next round of conjugational cross.

### Illumina sequencing preparation and analysis

The plasmid population pools isolated after 4th, 5th and 6th rounds of conjugational assay were selected and subjected to Illumina deep sequencing. Each pool of mutated *recA* gene was PCR-amplified with lower number of cycles and submitted to University of Wisconsin Biotechnology Center (UWBC) for amplicon library preparation and sequencing using the Illumina genome analyzer. Libraries were prepared for sequencing according to the manufacturer’s instructions with the following modifications. The initial input into each reaction was 100 ng of amplicon DNA, size selection procedure was omitted since library samples were single PCR products and PCR amplification was performed with 11 total cycles.

Data analysis was performed at the UWBC Bioinformatics Resource Center as following. Paired-end HiSeq data was merged using FastqJoin (http://code.google.com/p/ea-utils/wiki/FastqJoin). The un-joined reads were trimmed for low quality bases using the fastx toolkit (http://hannonlab.cshl.edu/fastx_toolkit) and joined by concatenating the reverse complement of reverse read to the end of forward read. The merged and joined sequences were then aligned to the *recA* sequence using the classic Smith—Waterman algorithm. The alignment adjusted for gaps and missing sequence data to produce a nucleotide counts by position summary. The unique reads were also counted and translated using the standard codon translation table. Sequences and the corresponding translations were evaluated for the variant and effects and then ranked according to the number of supporting combined reads.

### Electron microscopy (EM) experiments

A modified Alcian method was used to visualize RecA filaments on cssDNA. Activated grids were prepared as described previously [133]. All reactions were prepared by pre-incubating 3 μM RecA and 5 μM M13mp18 cssDNA, 25 mM Tris-OAc (80% cation) buffer, 5% (w/v) glycerol, 3 mM potassium glutamate, and 10 mM Mg (OAc)_2_. All reactions were carried out at 37°C. For an ATP regeneration system, 10 units/ml pyruvate kinase and 3.0 mM phosphoenolpyruvate were also added to pre-incubation mixture. After 10 min pre-incubation, 3 mM of ATP and 0.5 μM of SSB were added. After another 7 min, the RecX protein to a 100 nM or the equivalent volume of RecX storage buffer was added. ATPγS was then added to 3 mM, followed by 1 minute incubation. The reaction solution was then diluted to a final DNA concentration of 0.0004 μg/μl with 200 mm ammonium acetate, 10 mm HEPES (pH 7.5) and 10% glycerol and adsorbed onto Alcian grids for 3 min. The grid was then touched to a drop of the above buffer, followed by floating on a drop of the same buffer for 1 min. The sample was then stained by touching to a drop of 5% uranyl acetate followed by floating on a fresh drop of 5% uranyl acetate for 30 seconds. Finally, the grid was washed by touching to a drop of double distilled water followed by immersion in two 10 ml beakers of double distilled water. After the sample was dried, it was rotary-shadowed with platinum. This protocol is designed for visualization of complete reaction mixtures, and no attempt was made to remove unreacted material. Although this approach should yield results that provide insight into reaction components, it does lead to samples with a high background of unreacted proteins.

To determine the proportion of the molecules observed that were either fully or partially coated by RecA protein or bound only by the SSB protein, at least two separate regions of two to three independent experiments were counted at an identical magnification for each sample. "Full" filaments completely encompassed the circular DNA molecule or had small discontinuities in the regular striated pattern of the filament. A molecule was considered gapped if it had a detectable region of SSB-coated DNA of any size. Imaging and photography were carried out with a TECNAI G2 12 Twin Electron Microscope (FEI Co.) equipped with a GATAN 890 CCD camera. Digital images of the nucleoprotein filaments were taken at X 15,000 and X 26,000 magnification as is evident from the scale bar.

The observed lengths of the RecA filaments and the length of SSB-coated DNA were used to assign counted molecules to five categories: full filaments, medium filaments, small filaments, very small filaments or SSB/DNA molecules. Linearized DNA molecules, likely originating from shearing force during pipetting, were also counted. A RecA filament was considered a full filament if it does not have a detectable region of SSB coated DNA or a region that appeared to reduce the filament length by less than 10%. Medium filaments were smaller in length than full filaments, but still had substantial regions of nucleoprotein filament. Small filaments were generally less than half the length of full filaments, and often had regions of obvious SSB binding. Very small filamented molecules are those with just detectable segments of RecA filamented regions, with the rest of the molecule coated with SSB. With the total number of molecules counted as 100%, the percentage of each type of nucleoprotein filament was calculated. At least four separate regions of the grids encompassing at least 500 DNA molecules for each time point were counted at the identical magnification for each sample.

For each RecA variant, length measurements were carried out using Metamorph analysis software on 10 molecules selected at random from each of the five categories (excepting linears) that represented more than 10% of the total molecules in a given sample. In total, between 20 and 70 molecules from each of these five classes were measured, bound to the same ssDNA substrate. The complete set of measurements is provided in Table 2. Each filament was measured three times, and the average length was calculated. The 500 μm scale bar was used as a standard to calculate the number of pixels per μm. Each nucleoprotein fragment length, originally measured by Metamorph in pixels, was thus converted to μm.

### ATP hydrolysis (ATPase) assays

A coupled spectrophotometric enzyme assay [162, 163] was used to measure the DNA-dependent ATPase activities of the RecA protein. In this assay, the regeneration of ATP from ADP by pyruvate kinase and phosphoenolpyruvate was coupled to the oxidation of NADH by lactate dehydrogenase. The conversion of NADH to NAD^+^ was monitored as a decrease in absorbance at 380 nm rather than 340 nm, in order to remain in the linear range of the spectrophotometer for the duration of the experiment. The amount of ATP hydrolyzed over time was calculated using the NADH extinction coefficient at 380 nm of 1.21 mM^-1^cm^-1^. The assays were carried out on either a Varian Cary 300 dual beam spectrophotometer equipped with a temperature controller and a 12-position cell changer or Perkin Elmer Lambda 650UV/Vis spectrometer with 9+9 cell changer. The cell path length was 1.0 cm and the band pass was 2 nm. All reaction samples contained 25 mM Tris-OAc (80% cation, pH 7.4), 1 mM DTT, 3 mM potassium glutamate, 10 mM Mg(OAc)_2_, 5% (w/v) glycerol, an ATP regeneration system (10 units/ml pyruvate kinase and 3.0 mM phosphoenolpyruvate), 10 units/ml lactate dehydrogenase, 2.0 mM NADH, 5 M M13mp18 cssDNA or poly(dT) and 3 μM RecA proteins unless otherwise specified in the Fig legends.

### DNA three-strand exchange reaction experiments

DNA three-strand exchange reactions were carried out at 37°C in 25 mM Tris-OAc (80% cation, pH 7.4), 1 mM DTT, 3 mM potassium glutamate, 10 mM Mg(OAc)_2_, 5% (w/v) glycerol, an ATP regeneration system (10 units/ml pyruvate kinase and 2.0 mM phosphoenolpyruvate). The final pH after the addition of all reaction components was 7.4. The wild-type RecA protein and RecA mutant proteins (3.5 μM) were preincubated with 10 μM M13mp18 cssDNA for 10 min. The mixture of SSB protein (1 μM) and ATP (3 mM) was then added, followed by 10 min of incubation. DNA strand exchange reactions were initiated by the addition of M13mp18 lds (20 μM). Strand exchange reactions with EcRecX proteins were also carried out with the same concentration of DNA and proteins. For this reaction, RecX protein (0.1 μM) was added and incubated for 10 min before the reactions were initiated by adding ldsDNA. A 15 μl reaction aliquots were mixed with 5 μl of a solution containing 3 μl of Ficoll (0.4% bromophenol Blue, 0.4% xylene cyanol, 25% Ficoll, 120 mM EDTA) and 2 μl of 10% (w/v) SDS, and incubated for 40 min at 37°C to stop the reaction. Aliquots were loaded on a 0.8% agarose gel, and electrophoresed at 50 mA overnight at room temperature. The DNA was visualized by ethidium bromide staining and exposure to UV light. Gel images were captured with GE Typhoon FLA 9000 biomolecular imager and quantified using ImageQuant TL software from GE healthcare.

### D-loop forming reaction assays

D-loop forming reaction assays were carried out at 37°C in 25 mM Tris-OAc (80% cation, pH 7.4), 1 mM DTT, 3 mM potassium glutamate, 10 mM Mg(OAc)_2_, 5% (w/v) glycerol, an ATP regeneration system (10 units/ml pyruvate kinase and 2.0 mM phosphoenolpyruvate). The final pH after the addition of all reaction components was 7.4. The wild-type RecA protein and RecA mutant proteins (2 μM) were preincubated with 10 μM 3' overhung M13mp18 ldsDNA for 10 min. The mixture of SSB protein (1 μM) and ATP (3 mM) was then added, and incubated for an additional 10 min. The reactions were started by adding 10 μM M13mp18 cds. A 15 μl reaction aliquots were mixed with 5 μl of a solution containing 3 μl of Ficoll (0.4% bromophenol Blue, 0.4% xylene cyanol, 25% Ficoll, 120 mM EDTA) and 2 μl of 10% (w/v) SDS, and incubated for 40 min at 37°Cto stop the reaction. Aliquots were loaded on a 0.8% agarose gel, and electrophoresed at 50 mA overnight at room temperature. The DNA was visualized by ethidium bromide staining and exposure to UV light. Gel images were captured with GE Typhoon FLA 9000 biomolecular imager and quantified using ImageQuant TL software from GE healthcare.

### UV radiation and ciprofloxacin sensitivity tests

For UV irradiation sensitivity test, cells (EAW 105, 334, 394 and 410) were grown, serially diluted, and 100 μl of appropriate dilutions were spread onto LB plates. Dilutions for samples/treatments were empirically determined. The plates were then exposed to UV in a calibrated Spectrolinker XL-1000 UV crosslinker (Spectronics Corp) to the dose indicated. After incubating at 37°C overnight, the colonies were counted and divided by the dilution factor to get cfu/ml. For percent survival, colony counts on the treated plates were divided by the counts on untreated plates.

For ciprofloxacin experiments, plates were poured with LB agar containing the ciprofloxacin (0.01 μg/ml). Cells were grown, serially diluted, and spot plated (10 μl, 10^−2^ through 10^−6^) on the ciprofloxacin-containing plates. Pictures were taken after growing overnight at 37°C.

### Cell competition assays

Wild type cells, and in cells expressing any of several variant forms of RecA protein at the normal *recA* chromosomal locus, were modified to carry a neutral Ara^–^ mutation (which confers a red color on colonies when grown on tetrazolium arabinose (TA) indicator plates) to permit color based scoring of mixed populations [147]. Cells from a fresh single colony of each strain were cultured in LB broth [161] at 37°C with aeration. After growth overnight, competition cultures were started by inoculating 3 ml fresh LB broth with 30 μl of competition Ara+ or Ara^–^ strains and grown overnight at 37°C with shaking. Equal amounts of strains to be compared were mixed. A sample of the mixture was taken, diluted by a factor of 10^−6^, and plated on tetrazolium arabinose indicator plates. Then, 3 ml fresh LB broth was inoculated with 30 μl of the mixture, and grown overnight. The plating, inoculation, and growth cycle was repeated two more times. For experiments using cells containing plasmid pEAW947 (expressing NgRecX protein from the araBAD promoter), media was supplemented with 1% arabinose. White and red colonies were counted on plates containing 40–300 colonies, and the % of cells expressing mutant RecA proteins was determined. For counting colonies, plates with fewer than 20 colonies of either competitor were excluded to reduce the effect of outliers caused by low counts [164].

### SOS response assay

Overnight cultures were diluted 1:100 in fresh LB, and 200 μl was added to the wells of a black-walled, clear-bottom 96 well plate (Corning). For each sample, three overnights were grown from separate colonies, and each overnight filled three wells in the plate (three biological and three technical replicates, for nine total wells per sample). The plate was inserted into a Tecan infinite M1000 Pro plate reader. A program was used to incubate the plate at 37°C with orbital shaking. Every 10 min, the plate was briefly shaken linearly, and the OD_600_ and 509 nm emission (with 474 nm excitation) was read. SOS response was induced by adding ciprofloxacin (0.005 μg/ml) 3 hours after inoculation.

## Supporting Information

S1 Text Source code for confidence determination (in Python). (DOCX)

S1 Fig Characterization of the RecA mutant library. (A) Number of base substitutions in mutagenized *recA* genes and corresponding frequencies. Once recipient cells were transformed with the mutagenized *recA* plasmid pool, 42 randomly chosen single colony isolates were sequenced to estimate the actual *recA* mutation frequency. More than 70% of sequenced plasmids had no substitution, 16.6% had single substitutions, and sequences with more than two substitutions were below 5%. More than 27,500 single colonies, each transformed with a library plasmid, were combined together to make an initial cell library possessing more than 4,565 colonies (16.6% of the 27,500) with independent single base substitutions. (B) Determination of the number of colonies required to include all 531 single substitutions. A Monte Carlo simulation (see methods) was designed and run to determine the probability that our 4,565 mutant colonies included all 531 of the possible single substitutions that could occur within this 177 nucleotide (59 codon) region. The code was set up to choose a number at random from 1 to 531, and keep picking numbers until the entire set of numbers 1 to 531 was selected. The total number of random selections needed to accumulate the entire set of 531 was recorded for each trial, with each trial ending when the entire set of numbers was selected. One million trials were run using this code. “Number of trials requiring X mutants” is the number of total trials in which the number of random selections shown on the X axis was required to obtain all 531 possible mutations. The histogram shown details the output of these trials. This exercise defined the 90% confidence level as 4, 521 colonies, and the 95% confidence level as 4,904 colonies. With approximately 4,565 colonies with single base substitutions collected, our library meets the 90% confidence criterion.
(TIF)

S2 Fig Mutant to wild type RecA ratio as a function of selection cycle. The plasmid pools isolated from a broad sample of the pool of recombinant colonies generated after 4th, 5th and 6th round of conjugation were subjected to deep sequencing and summarized. The complete sequences were translated and placed in one of two categories, sequences with missense mutation and sequences with no or silent mutations.
(TIF)

S3 Fig Mutants detected after deep sequencing of the first directed evolution trial after the 4th, 5th and 6th cycle of selection. Heights of the bars reflect the percentage of the overall population represented by that mutant. The height of the light blue portion of each bar denotes the fraction of a particular mutant present as a single mutant. The medium blue and dark gray indicate the fraction of a particular mutation that were present as part of a double or triple mutant variant, respectively. All mutants representing more than 0.5% of the total *recA* genes are shown. Sequences from 4th, 5th and 6th round of conjugation in the first trial were translated to determine specific amino acid changes arising with prominence in the population. All amino acid changes which emerged from the 4th round of conjugation (V79L, E86G, C90G, I93L, H97Y, D100A, I102L and N113I) were consistently found through the 6th round of conjugation. The portion of the population with each of these mutations continued to increase with successive cycles except H97Y and N113I. Several amino acid changes (E127A, C129V, A131G, A131V, L132V and G136R) generated near the carboxyl-terminus of the mutated region appeared at detectable levels only after the 5th round of conjugation, and all occurred as part of double or triple mutants, such as E127A/A131G or C129V/A131V/L132V. The V79L and I102L single changes were the most prominent after every conjugation cycle, representing 7.5% and 10.8% of the population, respectively, after the 6th cycle of conjugation. The E86G/C90G double mutant was less than 2% of the population until the 5th conjugation cycle, but remarkably increased to 7.0% after the 6th round conjugation.
(TIF)

S4 Fig Mutations detected after deep sequencing of the second directed evolution trial after the 5th, 6th and 7th cycle of selection. Many of the same mutants were detected in both trials. Beginning with the original library, the entire selection procedure was repeated to determine reproducibility. A total of 7 cycles of selective conjugation were carried out in this 2nd selection experiment. The first three cycles were set up to require 4 crossovers, and the last four cycles required 6 crossovers. The amino acid changes found after 5th, 6th and 7th round of conjugation in this second selection experiment are shown in panel B. The I93L variant was most prominent after the seventh cycle (13.0% of the population), and the A131G variant was the second most prominent at 8.9%. The V79L and I102L changes that dominated the first experiment were 3.7% and 0.5% of the population, respectively. Bar coloring is as in S3 Fig.
(TIF)

S5 Fig The results after the final round of selection in both the 1st and 2nd directed evolution trials are aligned for comparison. Importantly, most of the RecA single mutants selected for in the second experiment were also found in the first experiment. The exceptions were limited to two variants (D100A and G136R) found only in the first experiment, and three others (A81V, A104V and D110A) found only in the second. The results suggest that the selection protocol is near saturation with respect to identifying RecA variants with improved recombination capacity in this region of the *recA* gene. Bar coloring is again as in S3 Fig.
(TIF)

S6 Fig Exchange of RecA variant protein between free and bound forms during three strand exchange reactions. RecA variant protein (0.8 μM) and M13mp18 ssDNA (2.4 μM) were incubated for 10 min to form nucleoprotein filament and ATP (3 mM) and SSB protein (0.24 μM) mixture was added to initiate reaction. The ATPase activity was monitored for 10 min before addition of the indicated amounts of RecA K72R mutant protein. RecA K72R addition (arrow) represented 0–80% of the concentration of the wild type RecA protein. The reaction was monitored another 20 min and M13mp18 ldsDNA (4.8 μM) was then added to initiate the DNA strand exchange reactions (second arrow). (A) ATP hydrolysis of wild type RecA protein during the reaction, (B) RecA V79L, (C) RecA I102L and RecA E86G/C90G and (D) wild type RecA and all variants with 0% or 80% K72R challenges. For the wild type RecA protein, the exchange of RecA subunits between free and bound forms is limited when RecA filaments are formed on closed circular ssDNA and SSB is added after RecA. The exchange between free and bound forms increases substantially when DNA strand exchange is initiated [42, 43, 114, 140]. This set of challenge experiments was carried out to assess RecA filament dynamics for the wild type and mutant proteins during strand exchange reactions. In this experiment, M13mp18 cssDNA was incubated with a stoichiometric concentration of either wild type or one of the selected mutant RecA proteins and ATP hydrolysis was initiated with addition of ATP and SSB protein mixture. After 10 min incubation, RecA K72R mutant protein, which binds but does not hydrolyze ATP [151], was added in amounts equivalent to 0%, 20%, 40%, 60% or 80% of the prebound RecA protein. After 20 min, the addition of M13 lds DNA was followed to trigger a strand exchange reaction. The RecA K72R mutant protein was used to detect RecA protomer exchange in the filament interior, as replacement of the bound RecA with the K72R mutant will lead to a decline in the measured ATPase [42]. The addition of RecA K72R at different levels prior to ldsDNA addition only slightly affected the rate of ATP hydrolysis as seen in the small decline during the last 7~8 min before adding ldsDNA, indicating a relatively low level of RecA protomer exchange. However, when homologous dsDNA was added to initiate strand exchange reaction, the rate of ATP hydrolysis noticeably declined due to the replacement of wild type protein with RecA K72R mutant protein [140]. The rates reported here reflect reaction velocities rather than apparent *k*_cat_ values. Panel A illustrates the immediate decline ATP hydrolysis rate of the wild type RecA protein proportional to the amount of added RecA K72R protein after the addition of M13 ldsDNA. At the highest level of added RecA K72R protein, the rate of ATP hydrolysis of wild type RecA protein declined by nearly 87% over the course of the 70 min time course. The rates seen with the RecA variants declined as well, but the declines were somewhat reduced, ranging from a 70.5% reduction by the RecA V79L variant (20.1 ± 2.6 μM/min (before adding lds) to 5.9 ± 2.0 μM/min (at 50~70 min) to an 84.4% reduction for the RecA E86G/C90G mutant (from 10.5 ± 4.9 to 1.6 ± 0.6 μM/min). The results suggest a modest reduction in filament dynamics during strand exchange for the RecA variants.
(TIF)

S7 Fig RecA-mediated DNA strand exchange by RecA variant proteins. RecA variant proteins were incubated with M13mp18 ssDNA to form nucleoprotein filaments and ATP hydrolysis began with addition of ATP and SSB protein mixtures. After another 10 min, three strand exchange reactions were initiated by adding M13mp18 ldsDNA. (A) The reactions were monitored by the agarose gel assay. The symbols mean: I, reaction intermediates; P, nicked circular DNA reaction products; S, linear duplex DNA substrates; and ss, circular ssDNA substrates. (B) Quantification of products and intermediates formed in the reactions. The capacity of the RecA variant proteins to promote DNA strand exchange was examined in this experiment. The reaction used is a standard assay in which RecA filaments formed on closed circular ssDNA promote strand exchange with homologous linear duplexes to yield a nicked circular duplex product. Branched DNA structures migrating above the product band in an agarose gel are intermediates in these reactions. As shown in panel A, the production of reaction intermediates was greater with the RecA variant proteins, particularly RecA I102L. However, those intermediates were converted to products more slowly than was the case with the wild type protein. The results suggest a modest reduction in the observed coupling between ATP hydrolysis and DNA strand exchange [43, 126, 128, 141, 142] in the variants.
(TIF)

S8 Fig D-loop formation promoted by RecA variant proteins at two different pHs. For the reaction, the RecA variant was incubated for 10 min with M13mp18 ldsDNA with 3′ extension on which the RecA protein was bound. ATP and SSB protein were added, and incubation continued for another 10 min before the addition of M13mp18 cdsDNA to initiate the reaction. The same reaction was carried out at both pH 7.5 and pH 8.8. (A) Agarose gel assay of reactions carried out in pH 7.5 buffer and quantification of D-loops formation. (B) Same reaction in pH 8.8 buffer and corresponding quantification. The decline in D-loops seen with time in some reactions reflects a D-loop cycle described by Radding and colleagues in the early 1980s [166, 167]. In this assay, RecA protein was first incubated with the M13mp18 linear dsDNA (ldsDNA) with a 3' single-stranded DNA extension to which the RecA bound, and then M13mp18 circular and supercoiled dsDNA (cdsDNA) was added to initiate reaction. RecA filament formation along the 3' tail led to strand invasion within a homologous cdsDNA, resulting in a D-loop. The wild type RecA began to form D-loops within 2 min and accumulated D-loop products up to 24.3 ± 1.1% after 15 min of reaction, followed by decrease of product. The RecA I102L mutant protein generated 25.5 ± 1.7% of final D-loop product at pH 7.5, suggesting little change in DNA pairing activity. The RecA V79L and RecA E86G/C90G mutant proteins exhibited somewhat lower D-loop forming activity than wild type RecA protein in that total amount of final products were 21.1 ± 3.6% and 21.0 ± 2.9%, respectively. The D-loop forming reaction assay was also carried out at higher pH, which was reported to inhibit RecA protein binding to dsDNA [37, 168, 169]. At pH 8.8, the D-loop forming activity of the wild type RecA protein declined by 56%. The reduction was substantially smaller for the RecA I102L and E86G/C90G mutant proteins, with 28.2% and 1.9% less D-loop product, respectively. The RecA V79L mutant protein produced a 10% gain in total product in pH 8.8, and promoted the reaction substantially better than the wild type protein. In general, the RecA variants exhibit a slightly enhanced DNA pairing activity, but only at high pH. It is not clear that this would translate into an activity advantage in the cell.
(TIF)

S9 Fig The effect of the RecX protein on three strand DNA exchange by RecA variant proteins. These experiments are identical to those in S7 Fig except for the addition of RecX protein (50 nM) at 7 min after the ATP and SSB were added and followed by 10 min incubation with RecX prior to the initiation of the strand exchange reaction. (A) Reactions monitored by the agarose gel assay. Symbols are as in S7 Fig legend. (B) Quantification of products and intermediates formed in the reactions. In this reaction, the wild type RecA protein began to form very small amounts of final products after 30 min incubation with the ldsDNA and only 2.4 ± 0.1% of total DNA substrates were resolved to final products after 100 min of reaction. For the RecA variants, some products appeared earlier, after 20 min reaction. Formation of intermediates also increased. Approximately 4.2~6.9% were transformed to final products. These results also indicate an improved capacity of the RecA variants to resist the inhibitory effects of RecX, although extensive strand exchange does exhibit substantial inhibition.
(TIF)

S10 Fig Conjugational recombination activity test for RecA V79L in the presence or absence of *recX* gene on the recipient chromosome. To investigate *in vivo* RecX function on conjugational recombination activity of wild type RecA protein and RecA V79L mutant protein, the *recX* gene was deleted (EAW537) from the recipient strain expressing wild type RecA and RecX protein(EAW174). A strain expressing RecA V79L variant (EAW530) was also tested in a Δ*recX* context (EAW542).
(TIF)
