# Supplementary material for: Directed Evolution of RecA Variants with Enhanced Capacity for Conjugational Recombination
Source: PLoS Genet. 2015 Jun 5;11(6):e1005278. doi: 10.1371/journal.pgen.1005278 (PMC4457935; doi:10.1371/journal.pgen.1005278)
Supplement: S1 Text — (DOCX) [file pgen.1005278.s001.docx]

**Supplementary Materials**

**Source code for confidence determination (in Python)**

import random

import math

########## ASSIGN A NUMBER OF TRIALS #########

num = 1000000

####### CREATE AN ARRAY TO STORE COUNTS ######

countarray = [0]*num

for i in range(num):

n = 0

count = 0

while n < 531:

########### PICK A RANDOM MUTATION ###########

mutant = random.randint(1,531)

##### DECIDE WHETHER THE MUTATION IS NEW #####

possible_new = 531 - n

## IF IT IS NEW, EDIT THE NUMBER OF POSSIBLE ##

if mutant <= possible_new:

n = n + 1

count = count + 1 #Count how many times have been tried

########## COUNT THE NUMBER OF PICKS #########

countarray[i] = count

####### BIN THE RESULTS FOR PLOTTING ######

binnum = max(countarray)

binarray = [0]*max(countarray)

for i in range(num):

val = countarray[i] - 1

binarray[val] += 1

########## PRINT MEAN AND MAX VALUES #########

print sum(countarray)/num

print max(countarray)

########### PRINT HISTOGRAM DATA #############

f = open('BioStats.txt', 'w')

for i in binarray:

print>>f, i

f.close

print 'ran'
