# Supplementary figures and images for: Directed Evolution of RecA Variants with Enhanced Capacity for Conjugational Recombination
Source: PLoS Genet. 2015 Jun 5;11(6):e1005278. doi: 10.1371/journal.pgen.1005278 (PMC4457935; doi:10.1371/journal.pgen.1005278)

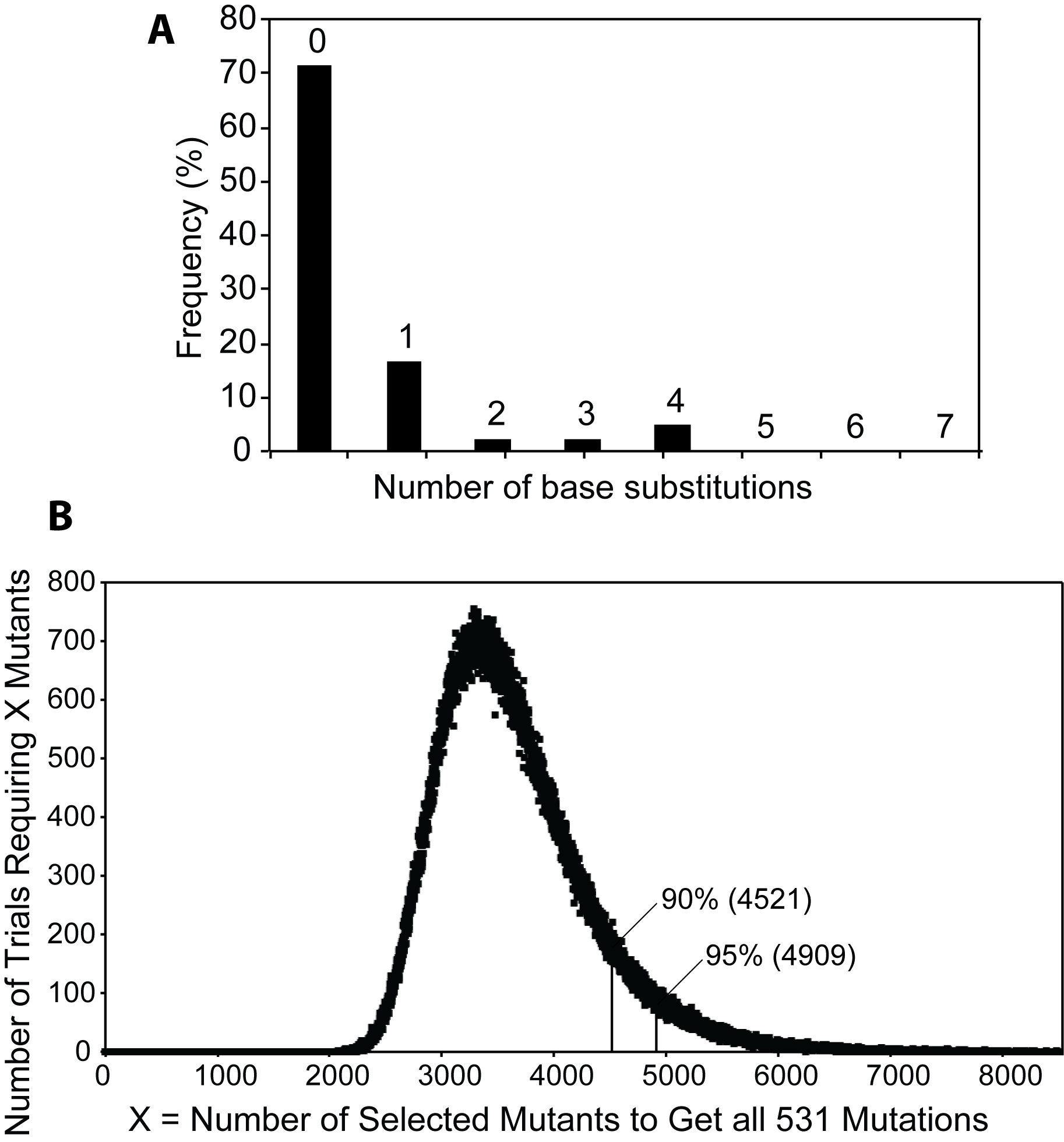

Supplement: S1 Fig — (A) Number of base substitutions in mutagenized recA genes and corresponding frequencies. Once recipient cells were transformed with the mutagenized recA plasmid pool, 42 randomly chosen single colony isolates were sequenced to estimate the actual recA mutation frequency. More than 70% of sequenced plasmids had no substitution, 16.6% had single substitutions, and sequences with more than two substitutions were below 5%. More than 27,500 single colonies, each transformed with a library plasmid, were combined together to make an initial cell library possessing more than 4,565 colonies (16.6% of the 27,500) with independent single base substitutions. (B) Determination of the number of colonies required to include all 531 single substitutions. A Monte Carlo simulation (see methods) was designed and run to determine the probability that our 4,565 mutant colonies included all 531 of the possible single substitutions that could occur within this 177 nucleotide (59 codon) region. The code was set up to choose a number at random from 1 to 531, and keep picking numbers until the entire set of numbers 1 to 531 was selected. The total number of random selections needed to accumulate the entire set of 531 was recorded for each trial, with each trial ending when the entire set of numbers was selected. One million trials were run using this code. “Number of trials requiring X mutants” is the number of total trials in which the number of random selections shown on the X axis was required to obtain all 531 possible mutations. The histogram shown details the output of these trials. This exercise defined the 90% confidence level as 4, 521 colonies, and the 95% confidence level as 4,904 colonies. With approximately 4,565 colonies with single base substitutions collected, our library meets the 90% confidence criterion. (TIF) [file pgen.1005278.s002.tif]

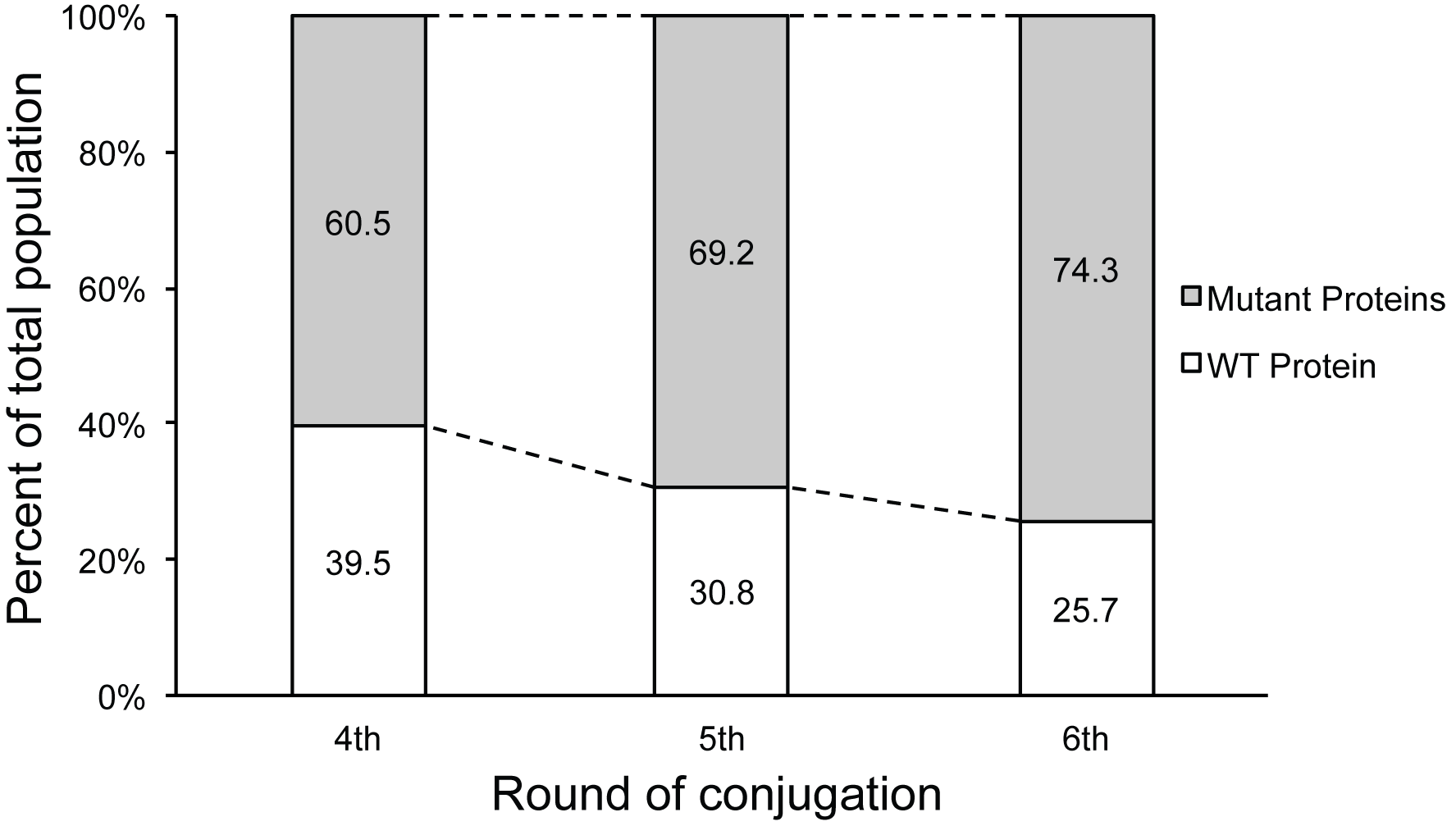

Supplement: S2 Fig — The plasmid pools isolated from a broad sample of the pool of recombinant colonies generated after 4th, 5th and 6th round of conjugation were subjected to deep sequencing and summarized. The complete sequences were translated and placed in one of two categories, sequences with missense mutation and sequences with no or silent mutations. (TIF) [file pgen.1005278.s003.tif]

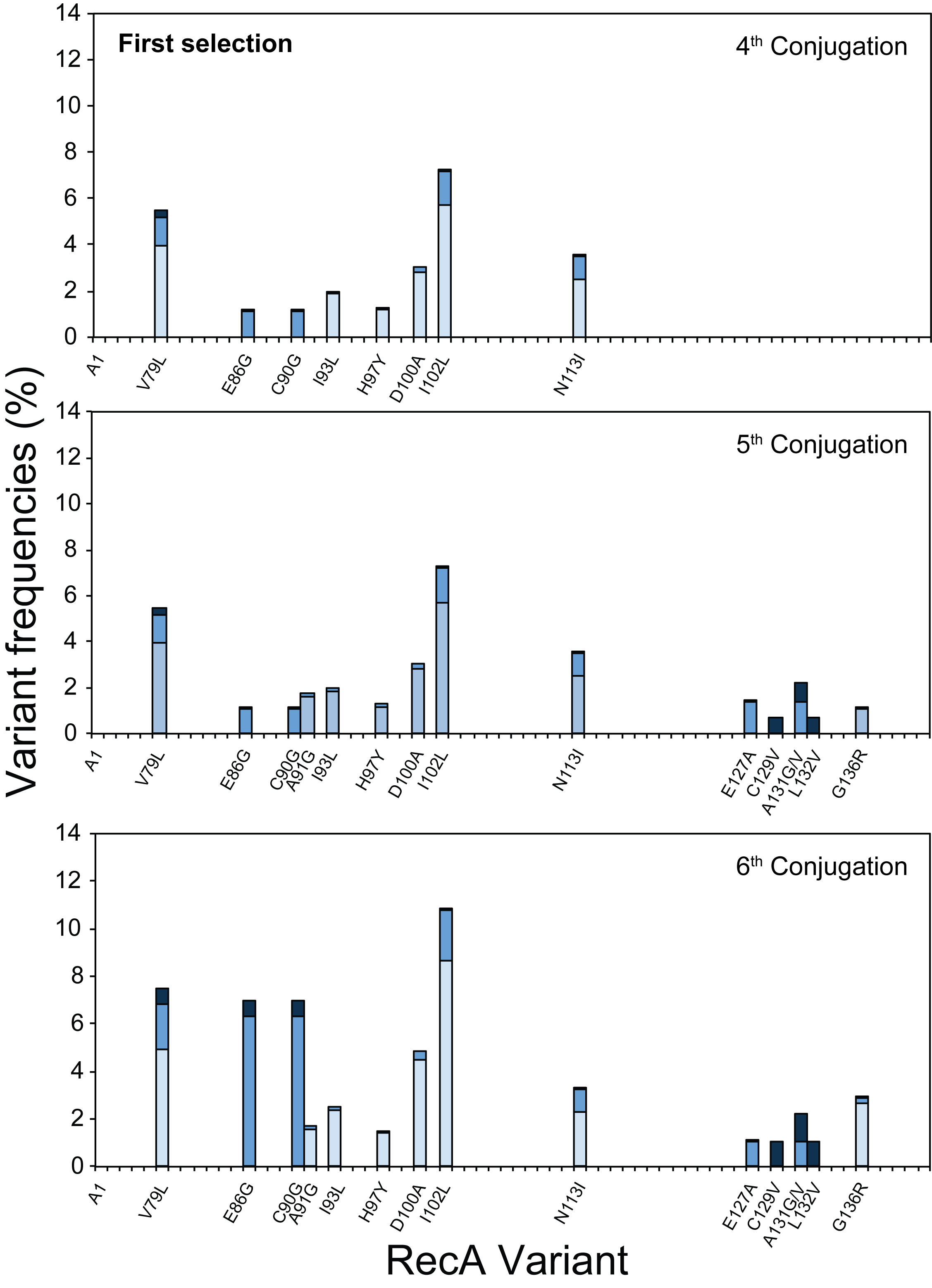

Supplement: S3 Fig — Heights of the bars reflect the percentage of the overall population represented by that mutant. The height of the light blue portion of each bar denotes the fraction of a particular mutant present as a single mutant. The medium blue and dark gray indicate the fraction of a particular mutation that were present as part of a double or triple mutant variant, respectively. All mutants representing more than 0.5% of the total recA genes are shown. Sequences from 4th, 5th and 6th round of conjugation in the first trial were translated to determine specific amino acid changes arising with prominence in the population. All amino acid changes which emerged from the 4th round of conjugation (V79L, E86G, C90G, I93L, H97Y, D100A, I102L and N113I) were consistently found through the 6th round of conjugation. The portion of the population with each of these mutations continued to increase with successive cycles except H97Y and N113I. Several amino acid changes (E127A, C129V, A131G, A131V, L132V and G136R) generated near the carboxyl-terminus of the mutated region appeared at detectable levels only after the 5th round of conjugation, and all occurred as part of double or triple mutants, such as E127A/A131G or C129V/A131V/L132V. The V79L and I102L single changes were the most prominent after every conjugation cycle, representing 7.5% and 10.8% of the population, respectively, after the 6th cycle of conjugation. The E86G/C90G double mutant was less than 2% of the population until the 5th conjugation cycle, but remarkably increased to 7.0% after the 6th round conjugation. (TIF) [file pgen.1005278.s004.tif]

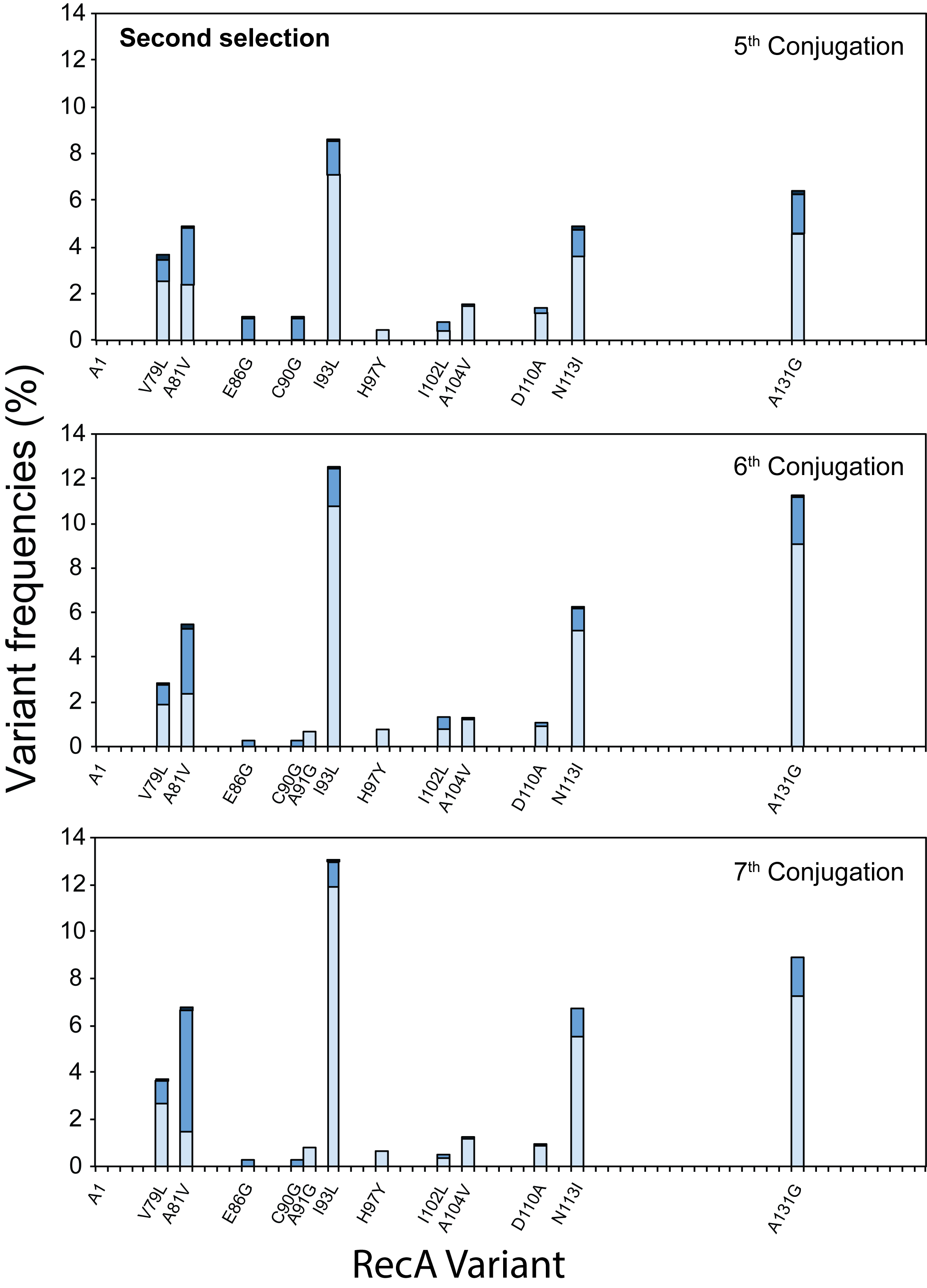

Supplement: S4 Fig — Many of the same mutants were detected in both trials. Beginning with the original library, the entire selection procedure was repeated to determine reproducibility. A total of 7 cycles of selective conjugation were carried out in this 2nd selection experiment. The first three cycles were set up to require 4 crossovers, and the last four cycles required 6 crossovers. The amino acid changes found after 5th, 6th and 7th round of conjugation in this second selection experiment are shown in panel B. The I93L variant was most prominent after the seventh cycle (13.0% of the population), and the A131G variant was the second most prominent at 8.9%. The V79L and I102L changes that dominated the first experiment were 3.7% and 0.5% of the population, respectively. Bar coloring is as in S3 Fig. (TIF) [file pgen.1005278.s005.tif]

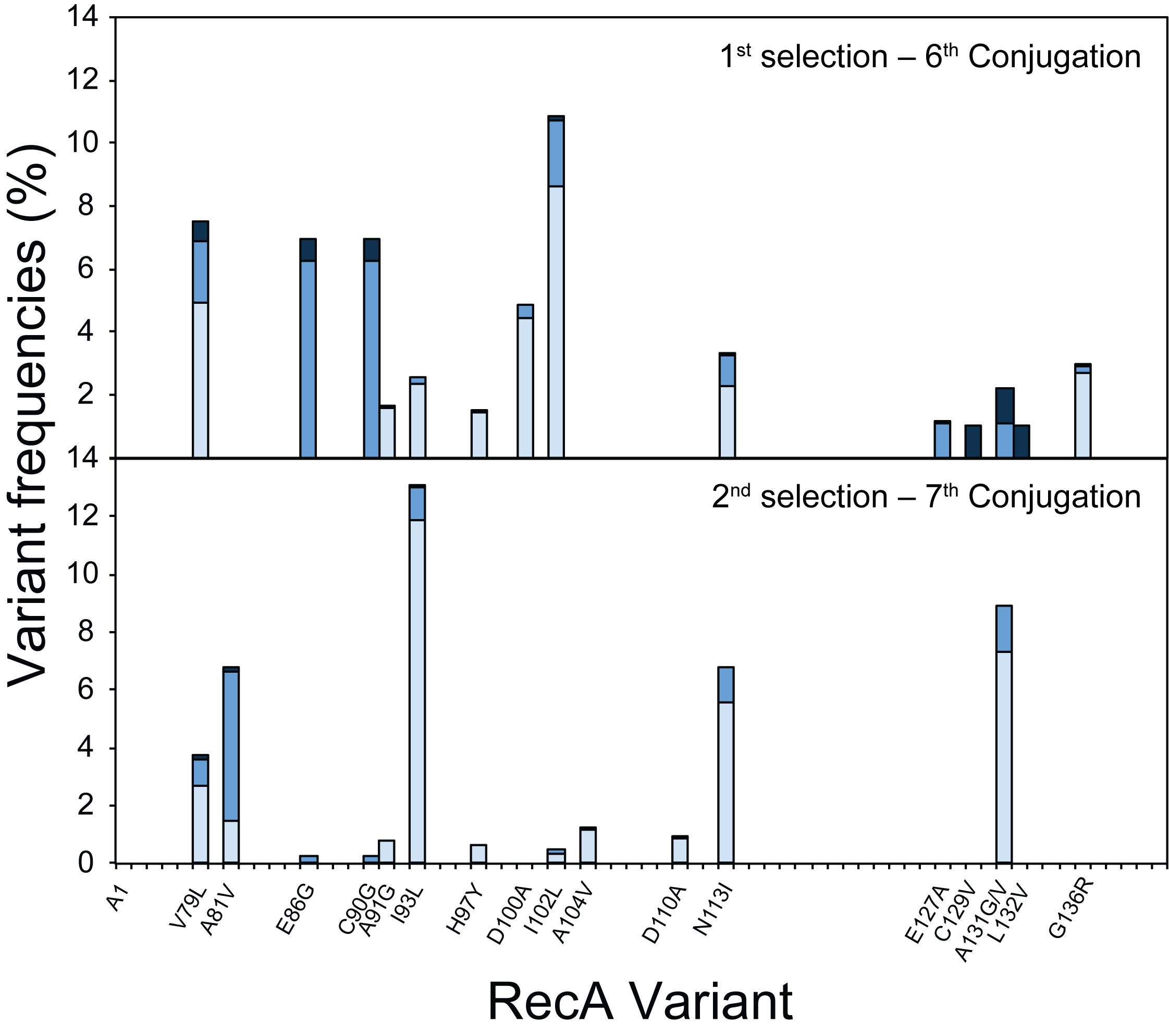

Supplement: S5 Fig — Importantly, most of the RecA single mutants selected for in the second experiment were also found in the first experiment. The exceptions were limited to two variants (D100A and G136R) found only in the first experiment, and three others (A81V, A104V and D110A) found only in the second. The results suggest that the selection protocol is near saturation with respect to identifying RecA variants with improved recombination capacity in this region of the recA gene. Bar coloring is again as in S3 Fig. (TIF) [file pgen.1005278.s006.tif]

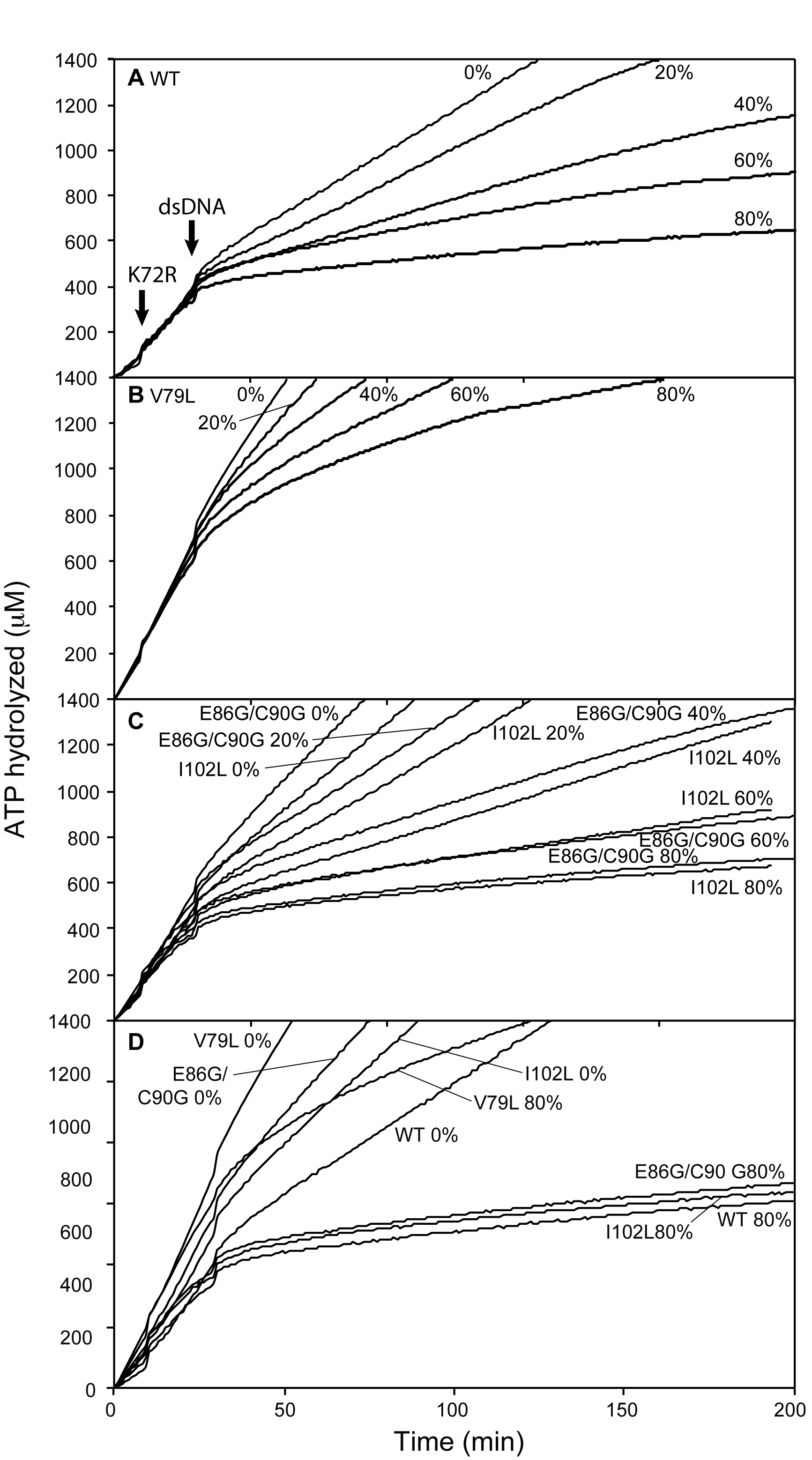

Supplement: S6 Fig — RecA variant protein (0.8 μM) and M13mp18 ssDNA (2.4 μM) were incubated for 10 min to form nucleoprotein filament and ATP (3 mM) and SSB protein (0.24 μM) mixture was added to initiate reaction. The ATPase activity was monitored for 10 min before addition of the indicated amounts of RecA K72R mutant protein. RecA K72R addition (arrow) represented 0–80% of the concentration of the wild type RecA protein. The reaction was monitored another 20 min and M13mp18 ldsDNA (4.8 μM) was then added to initiate the DNA strand exchange reactions (second arrow). (A) ATP hydrolysis of wild type RecA protein during the reaction, (B) RecA V79L, (C) RecA I102L and RecA E86G/C90G and (D) wild type RecA and all variants with 0% or 80% K72R challenges. For the wild type RecA protein, the exchange of RecA subunits between free and bound forms is limited when RecA filaments are formed on closed circular ssDNA and SSB is added after RecA. The exchange between free and bound forms increases substantially when DNA strand exchange is initiated [42, 43, 114, 140]. This set of challenge experiments was carried out to assess RecA filament dynamics for the wild type and mutant proteins during strand exchange reactions. In this experiment, M13mp18 cssDNA was incubated with a stoichiometric concentration of either wild type or one of the selected mutant RecA proteins and ATP hydrolysis was initiated with addition of ATP and SSB protein mixture. After 10 min incubation, RecA K72R mutant protein, which binds but does not hydrolyze ATP [151], was added in amounts equivalent to 0%, 20%, 40%, 60% or 80% of the prebound RecA protein. After 20 min, the addition of M13 lds DNA was followed to trigger a strand exchange reaction. The RecA K72R mutant protein was used to detect RecA protomer exchange in the filament interior, as replacement of the bound RecA with the K72R mutant will lead to a decline in the measured ATPase [42]. The addition of RecA K72R at different levels prior to ldsDNA addition only sligh [file pgen.1005278.s007.tif]

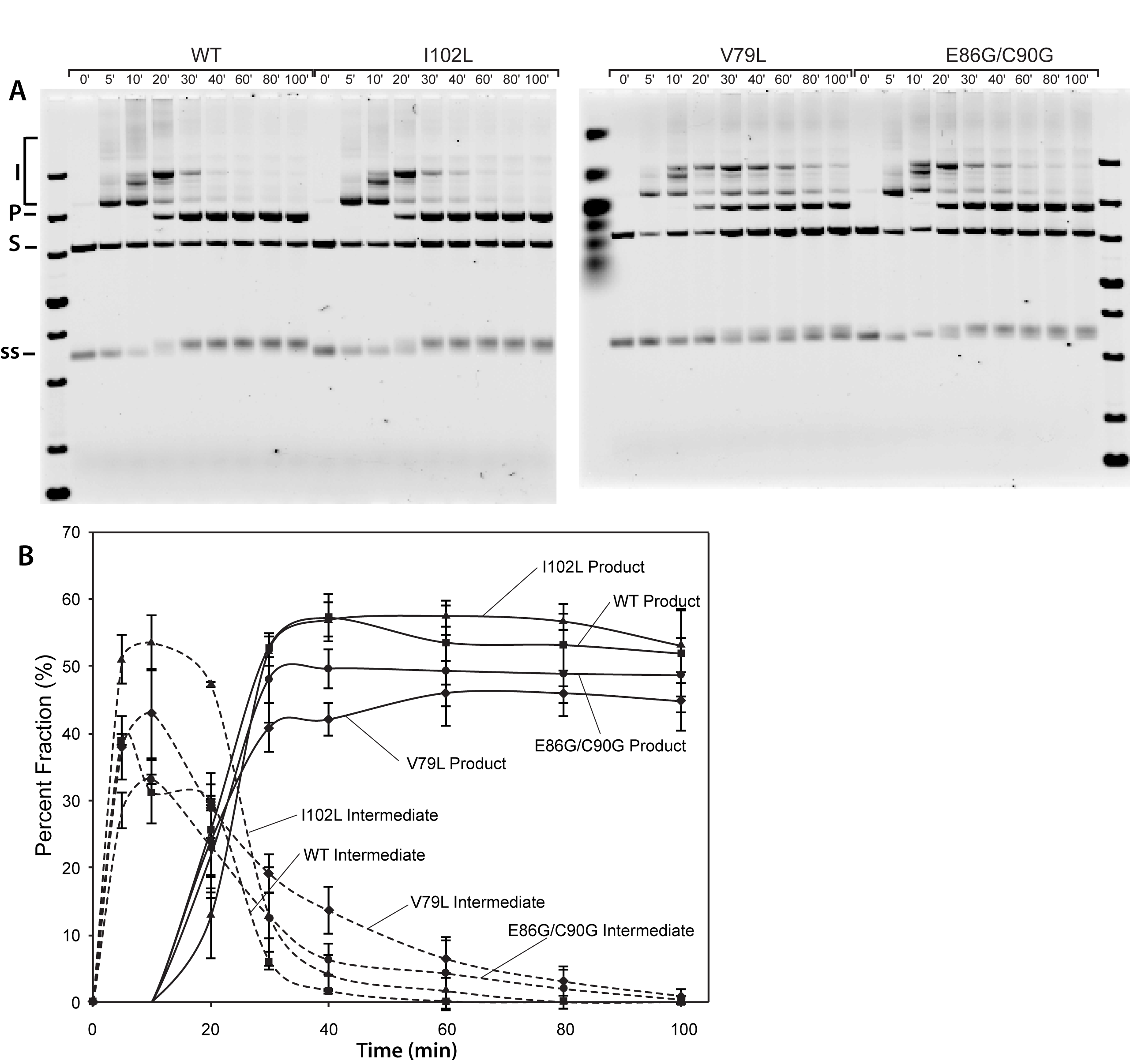

Supplement: S7 Fig — RecA variant proteins were incubated with M13mp18 ssDNA to form nucleoprotein filaments and ATP hydrolysis began with addition of ATP and SSB protein mixtures. After another 10 min, three strand exchange reactions were initiated by adding M13mp18 ldsDNA. (A) The reactions were monitored by the agarose gel assay. The symbols mean: I, reaction intermediates; P, nicked circular DNA reaction products; S, linear duplex DNA substrates; and ss, circular ssDNA substrates. (B) Quantification of products and intermediates formed in the reactions. The capacity of the RecA variant proteins to promote DNA strand exchange was examined in this experiment. The reaction used is a standard assay in which RecA filaments formed on closed circular ssDNA promote strand exchange with homologous linear duplexes to yield a nicked circular duplex product. Branched DNA structures migrating above the product band in an agarose gel are intermediates in these reactions. As shown in panel A, the production of reaction intermediates was greater with the RecA variant proteins, particularly RecA I102L. However, those intermediates were converted to products more slowly than was the case with the wild type protein. The results suggest a modest reduction in the observed coupling between ATP hydrolysis and DNA strand exchange [43, 126, 128, 141, 142] in the variants. (TIF) [file pgen.1005278.s008.tif]

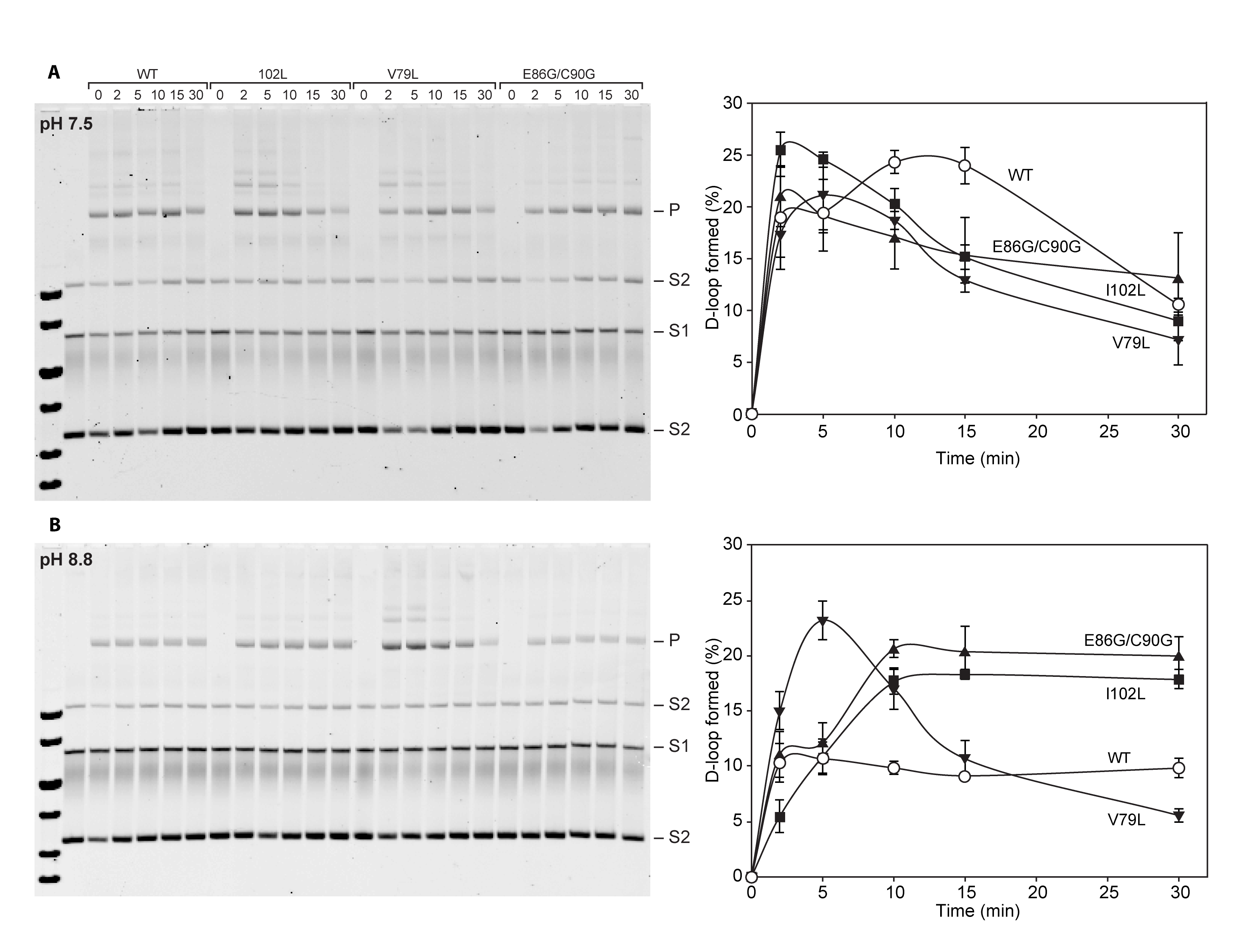

Supplement: S8 Fig — For the reaction, the RecA variant was incubated for 10 min with M13mp18 ldsDNA with 3′ extension on which the RecA protein was bound. ATP and SSB protein were added, and incubation continued for another 10 min before the addition of M13mp18 cdsDNA to initiate the reaction. The same reaction was carried out at both pH 7.5 and pH 8.8. (A) Agarose gel assay of reactions carried out in pH 7.5 buffer and quantification of D-loops formation. (B) Same reaction in pH 8.8 buffer and corresponding quantification. The decline in D-loops seen with time in some reactions reflects a D-loop cycle described by Radding and colleagues in the early 1980s [166, 167]. In this assay, RecA protein was first incubated with the M13mp18 linear dsDNA (ldsDNA) with a 3' single-stranded DNA extension to which the RecA bound, and then M13mp18 circular and supercoiled dsDNA (cdsDNA) was added to initiate reaction. RecA filament formation along the 3' tail led to strand invasion within a homologous cdsDNA, resulting in a D-loop. The wild type RecA began to form D-loops within 2 min and accumulated D-loop products up to 24.3 ± 1.1% after 15 min of reaction, followed by decrease of product. The RecA I102L mutant protein generated 25.5 ± 1.7% of final D-loop product at pH 7.5, suggesting little change in DNA pairing activity. The RecA V79L and RecA E86G/C90G mutant proteins exhibited somewhat lower D-loop forming activity than wild type RecA protein in that total amount of final products were 21.1 ± 3.6% and 21.0 ± 2.9%, respectively. The D-loop forming reaction assay was also carried out at higher pH, which was reported to inhibit RecA protein binding to dsDNA [37, 168, 169]. At pH 8.8, the D-loop forming activity of the wild type RecA protein declined by 56%. The reduction was substantially smaller for the RecA I102L and E86G/C90G mutant proteins, with 28.2% and 1.9% less D-loop product, respectively. The RecA V79L mutant protein produced a 10% gain in total product in pH 8.8, and promoted the rea [file pgen.1005278.s009.tif]

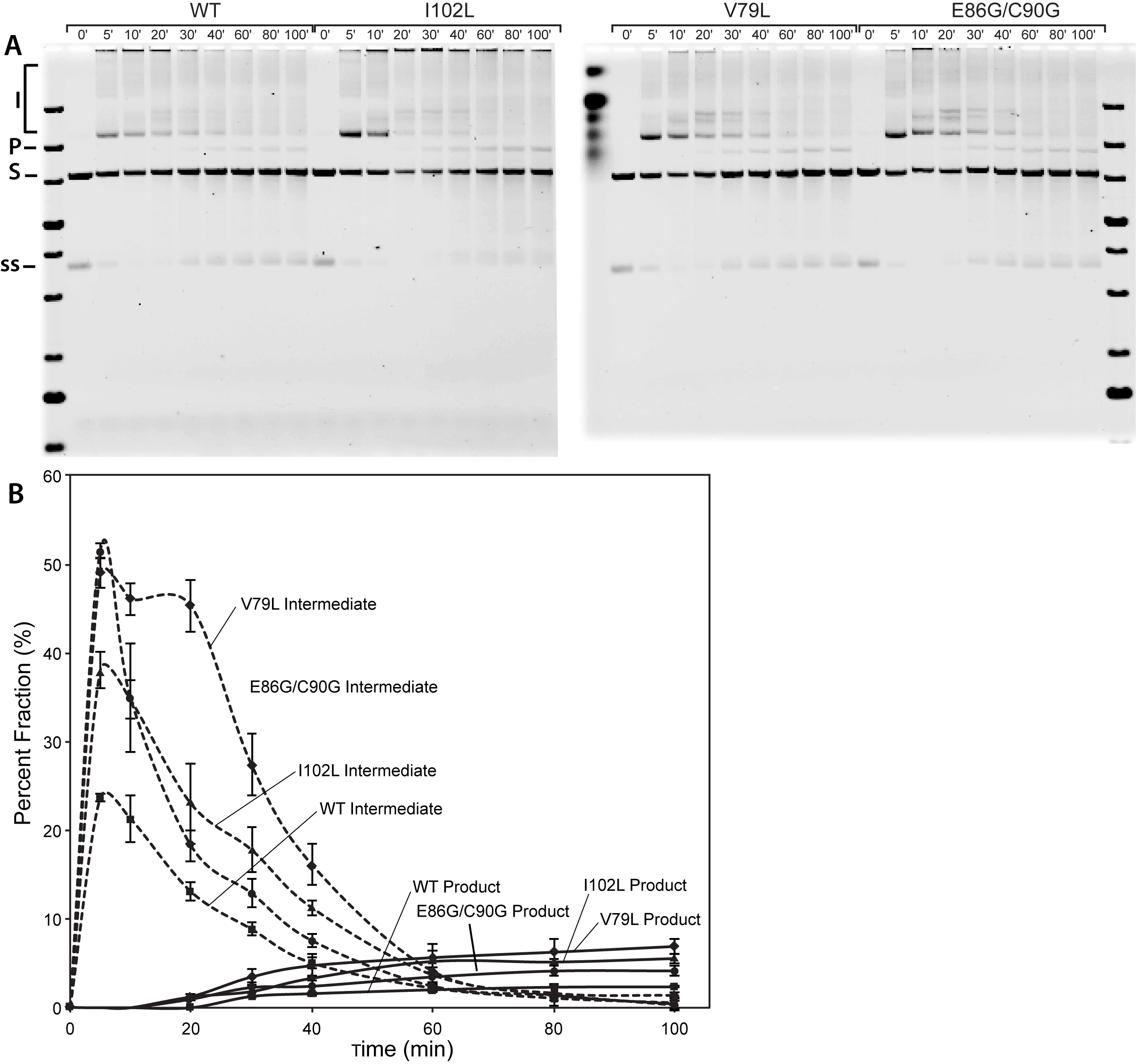

Supplement: S9 Fig — These experiments are identical to those in S7 Fig except for the addition of RecX protein (50 nM) at 7 min after the ATP and SSB were added and followed by 10 min incubation with RecX prior to the initiation of the strand exchange reaction. (A) Reactions monitored by the agarose gel assay. Symbols are as in S7 Fig legend. (B) Quantification of products and intermediates formed in the reactions. In this reaction, the wild type RecA protein began to form very small amounts of final products after 30 min incubation with the ldsDNA and only 2.4 ± 0.1% of total DNA substrates were resolved to final products after 100 min of reaction. For the RecA variants, some products appeared earlier, after 20 min reaction. Formation of intermediates also increased. Approximately 4.2~6.9% were transformed to final products. These results also indicate an improved capacity of the RecA variants to resist the inhibitory effects of RecX, although extensive strand exchange does exhibit substantial inhibition. (TIF) [file pgen.1005278.s010.tif]

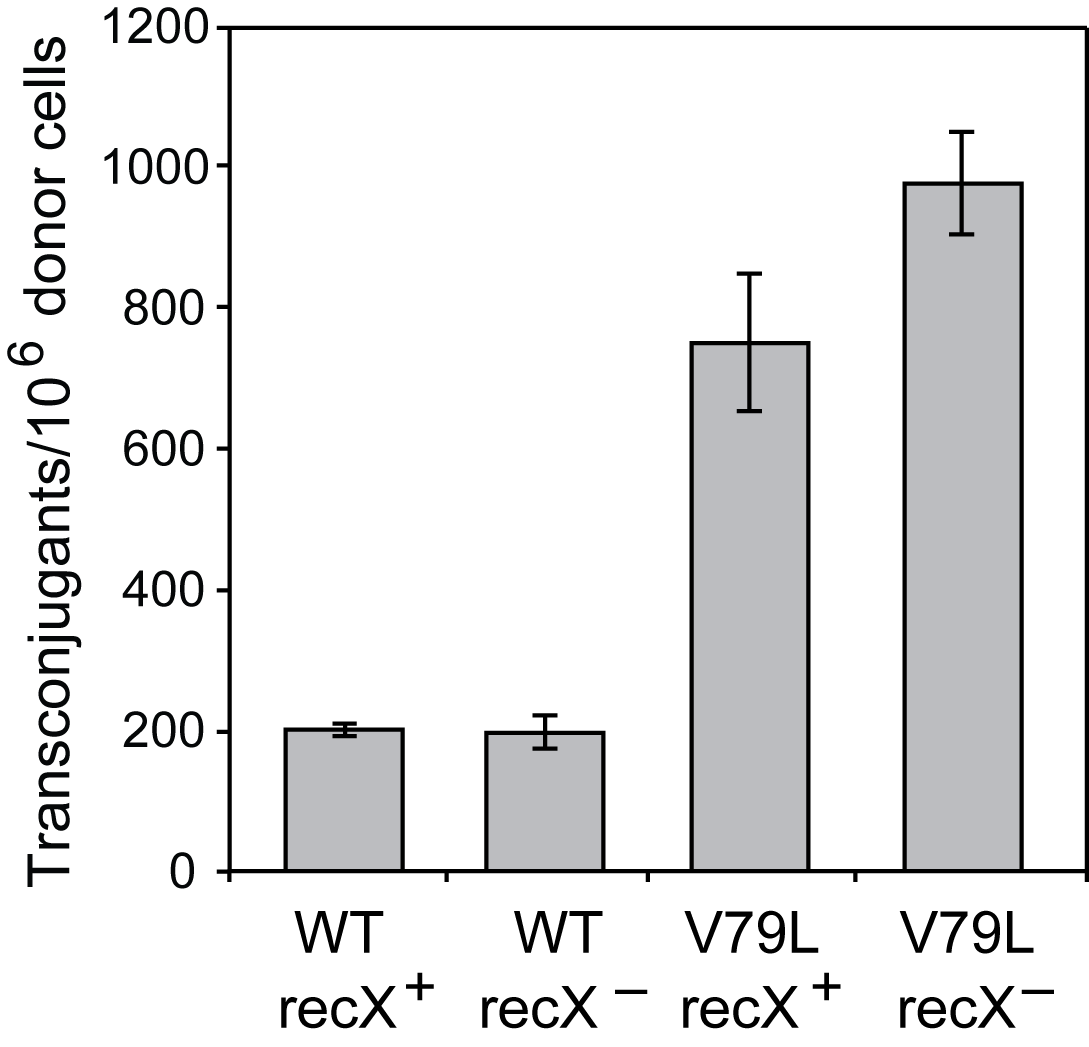

Supplement: S10 Fig — To investigate in vivo RecX function on conjugational recombination activity of wild type RecA protein and RecA V79L mutant protein, the recX gene was deleted (EAW537) from the recipient strain expressing wild type RecA and RecX protein(EAW174). A strain expressing RecA V79L variant (EAW530) was also tested in a ΔrecX context (EAW542). (TIF) [file pgen.1005278.s011.tif]
